# Exploring Electrocortical Signatures of Gait Adaptation: Differential Neural Dynamics in Slow and Fast Gait Adapters

**DOI:** 10.1523/ENEURO.0515-23.2024

**Published:** 2024-07-04

**Authors:** Noelle A. Jacobsen, Daniel P. Ferris

**Affiliations:** J. Crayton Pruitt Family Department of Biomedical Engineering, University of Florida, Gainesville, Florida 32611-6131

**Keywords:** brain, electroencephalography, human, locomotion, motor adaptation

## Abstract

Individuals exhibit significant variability in their ability to adapt locomotor skills, with some adapting quickly and others more slowly. Differences in brain activity likely contribute to this variability, but direct neural evidence is lacking. We investigated individual differences in electrocortical activity that led to faster locomotor adaptation rates. We recorded high-density electroencephalography while young, neurotypical adults adapted their walking on a split-belt treadmill and grouped them based on how quickly they restored their gait symmetry. Results revealed unique spectral signatures within the posterior parietal, bilateral sensorimotor, and right visual cortices that differ between fast and slow adapters. Specifically, fast adapters exhibited lower alpha power in the posterior parietal and right visual cortices during early adaptation, associated with quicker attainment of steady-state step length symmetry. Decreased posterior parietal alpha may reflect enhanced spatial attention, sensory integration, and movement planning to facilitate faster locomotor adaptation. Conversely, slow adapters displayed greater alpha and beta power in the right visual cortex during late adaptation, suggesting potential differences in visuospatial processing. Additionally, fast adapters demonstrated reduced spectral power in the bilateral sensorimotor cortices compared with slow adapters, particularly in the theta band, which may suggest variations in perception of the split-belt perturbation. These findings suggest that alpha and beta oscillations in the posterior parietal and visual cortices and theta oscillations in the sensorimotor cortex are related to the rate of gait adaptation.

## Graphical abstract

**Figure:**
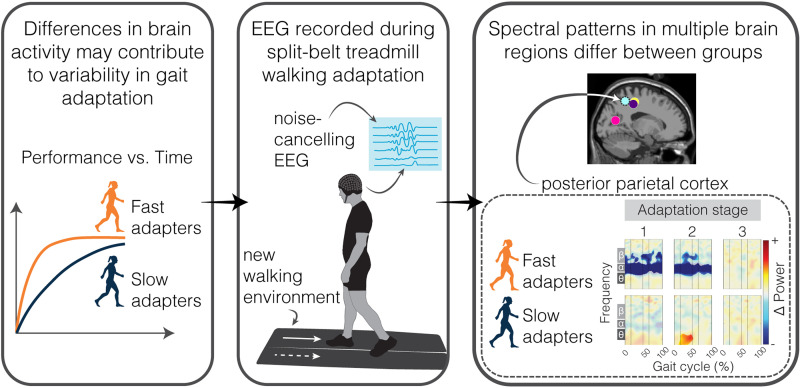
Visual Overview

## Significance Statement

The specific neural dynamics and factors influencing variability in individual locomotor adaptation rates remain active areas of exploration. We provide a novel characterization of cortical dynamics associated with gait adaptability in young, neurotypical adults. We identified distinct electrocortical patterns in the posterior parietal, sensorimotor, and visual cortices that differ between those who adapt to a split-belt treadmill quickly versus slowly. Notably, our findings suggest that posterior parietal alpha plays a crucial role in enhancing locomotor adaptation, potentially by regulating multisensory integration and visuospatial attention.

## Introduction

Individuals display significant variability in their ability to acquire new locomotor skills. Some adapt quickly, efficiently recalibrating their motor patterns, while others progress more slowly, requiring additional time and practice. Age also plays a role, with young children (Vasudevan et al., 2011) and older adults (Bruijn et al., 2012) showing slower rates of gait adaptation compared with young adults. Additionally, neurological damage can hinder locomotor adaptation (Dietz et al., 1995; Morton and Bastian, 2006; Choi et al., 2009; Malone and Bastian, 2014; Vasudevan et al., 2014; Mawase et al., 2016), which not only reduces mobility but also increases fall risk. These pronounced differences in adaptation rates, seen in both neurotypical and neurologically impaired individuals, have spurred research into the biomechanical and neural sources of this variability.

Previous research has demonstrated that individual differences in the rate of sensorimotor adaptation are related to variability in task-related brain activity and perception. Individual differences in visuomotor adaptation have been linked to differences in proprioception (Tsay et al., 2021), spatial working memory (Anguera et al., 2010), brain structure (Della-Maggiore et al., 2009; Donchin et al., 2012), and brain activity (Anguera et al., 2010, 2011; Della-Maggiore et al., 2017; Ruitenberg et al., 2018). Functional magnetic resonance imaging has shown that activation levels of the right dorsolateral prefrontal (Anguera et al., 2011), cingulate, visual, and parietal cortices (Seidler et al., 2006) predicted performance during early visuomotor adaptation. The more participants recruited these regions, the faster they adapted. However, most studies investigating neural correlates of adaptation have focused on discrete upper-limb tasks, such as force-field reaching (Vahdat et al., 2011, 2014; Moore and Cluff, 2021) or joystick movements (Anguera et al., 2010). There is limited knowledge about neural correlates of adaptation during continuous whole-body tasks such as walking, despite the significance of such tasks in daily life, sports, and rehabilitation.

To gain insights on neural control of locomotor adaptation, many researchers have studied humans walking during tasks that require compensation for systematic, sensory, or mechanical perturbations. One widely used tool for investigating gait adaptation is a split-belt treadmill, which challenges individuals to alter their leg movements to account for two different belt speeds. To reduce step length asymmetry while walking on a split-belt treadmill, participants must adjust their leg trajectory. After a short adaptation session, participants learn to lengthen steps with the fast leg and shorten those with the slow leg. Although many split-belt studies have inferred neural underpinnings of gait adaptation from kinetic, kinematic, electromyographic, and clinical data, documentation of associated brain dynamics has been limited.

The small body of work on brain imaging during locomotor adaptation suggests a distributed network of brain regions are involved in adaptation. Locomotor adaptation requires a comprehensive understanding of body orientation, spatial motion, and motion perception. This is achieved through the integration of vestibular, somatosensory, and visual sensory signals in both the cerebral cortex and cerebellum. Studies have indicated that multiple cortical regions, including the sensorimotor, cingulate, and parietal cortices, are engaged during gait adaptation in response to a split-belt perturbation (Hinton et al., 2019; Jacobsen and Ferris, 2023) and auditory cues (Wagner et al., 2016, 2019). These cortical regions likely work in concert with the cerebellum to process sensory feedback and fine-tune motor commands. Variations in adaptation rate (Vasudevan et al., 2011; Bruijn et al., 2012; Rashid et al., 2020; Jacobsen et al., 2023), retention (Roemmich and Bastian, 2015), and generalization (Torres-Oviedo and Bastian, 2012; Mariscal et al., 2020) of new gait patterns suggest underlying neural disparities that are largely uncharted. One reason for this is that brain imaging techniques such as functional magnetic resonance imaging require participants to be stationary.

Quantifying brain activity during locomotor adaptation could reveal neural signatures linked to individual differences in adaptation rates, paving the way for future research on populations with gait impairments. If there is significant variability in individual neural responses to identical locomotor challenges, these insights could aid in facilitating performance for individuals struggling to adapt to new locomotor patterns. Additionally, investigating the variability in brain activity among neurotypical individuals may improve our ability to characterize abnormal patterns within clinical populations. Integrating neurofeedback into rehabilitation protocols could yield valuable neural predictors of therapy response and enable real-time adjustments, potentially enhancing treatment outcomes. Modulation of electrocortical activity through brain stimulation has the potential to improve motor task learning (Byczynski and Vanneste, 2023); however, current research indicates high interindividual variability in response to various types of brain stimulation [reviewed in Byczynski and Vanneste (2023); Krause and Kadosh, 2014; López-Alonso et al., 2014; Buch et al., 2017]. Therefore, further exploration of the interindividual variability of electrocortical correlates of gait adaptation could significantly advance personalized interventions for individuals with gait impairments.

The posterior parietal cortex plays a key role in sensorimotor adaptation, particularly in tasks such as split-belt treadmill adaptation. This brain region is classically thought to be crucial for spatial attention (Verfaellie et al., 1990; Steinmetz and Constantinidis, 1995; Friedrich et al., 1998), multimodal integration (Kerkhoff, 1999; Lewis and Van Essen, 2000), and movement planning [reviewed in Andersen et al. (2003)]. Sensory signals and efference copies from motor regions converge in the posterior parietal cortex to help plan movements. Additionally, higher cognitive functions, including attention, occur within this region [reviewed in Andersen et al. (2003)]. Early adaptive processes have been associated with increased blood oxygenation in posterior parietal regions during visuomotor adaptation using a joystick (Anguera et al., 2007). In the context of human locomotion, evidence suggests that the posterior parietal cortex is involved in novel or challenging gait tasks (An et al., 2019; Hinton et al., 2019; Nordin et al., 2019; Young et al., 2020). Inhibition of the posterior parietal cortex via transcranial magnetic simulation has been shown to decrease adaptation rate during split-belt walking (Young et al., 2020). While the posterior parietal cortex receives less attention compared with the motor, premotor, supplementary, and cerebellar cortices, it holds potential as a target for enhancing motor learning through noninvasive brain stimulation. Given the posterior parietal cortex's involvement in sensorimotor adaptation, movement planning, and attention, neural activity in this region may differ between fast and slow gait adapters.

Recent advances in high-density electroencephalography (EEG) techniques allow researchers to measure human electrocortical activity during locomotion, even in the presence of motion and muscle artifacts [for a comprehensive overview, refer to Song and Nordin (2021)]. EEG, with its high temporal resolution, can track gait-related neural activity throughout adaptation. We quantified electrocortical activity during locomotor adaptation to a split-belt treadmill perturbation and examined differences in gait-related spectral power between two groups, sorted based on how quickly they restored their gait symmetry. Evidence from EEG recorded during locomotor adaptation suggests that alpha (8–12 Hz), beta (13–30 Hz), and theta (4–7 Hz) oscillations may be related to motor error monitoring (Sipp et al., 2013; Tan et al., 2014), standing postural stability (Solis-Escalante et al., 2021; Stokkermans et al., 2022), and balance perturbations during walking (Peterson and Ferris, 2018). Gait-related spectral perturbation data from the posterior parietal, sensorimotor, and anterior cingulate cortices under the same conditions have been published previously. These findings were derived from a combined participant group and can be found in Jacobsen and Ferris (2023). Our results indicated that sensorimotor and posterior parietal cortices had decreased alpha and beta power during early adaptation to split-belt treadmill walking that gradually dissipated by the end of the adaptation period. Additionally, we observed increased theta within the anterior cingulate at the beginning stages of adaptation. In this current study, we partitioned our study population into two subgroups based on their rate of adaptation. Additionally, we incorporated two newly added cortical clusters located near the left and right visual cortex into our analysis. We hypothesized that spectral power analyses would reveal differences in electrocortical activity in motor and sensory brain regions between fast adapters and slow adapters.

## Materials and Methods

Between this section and the following results section, we have reported how we determined our sample size, all data exclusions (if any), all manipulations, and all measures in the study.

### Participants

We recruited a total of 33 neurotypical, young adults (15 females, 18 males) for our study. Our power analysis (effect size *f* = 0.39) on pilot data for the alpha band informed us that 21 participants would be required for sufficient power [90%; G*Power (Faul et al., 2007)]. Given the likelihood that not every cortical cluster would have a representative participant and a couple participants would potentially be removed because of equipment or data quality issues, we recruited an additional 12 participants. The experimental protocol was approved by the University of Florida Institutional Review Board (IRB201701603) and adhered to the principles outlined in the Declaration of Helsinki. All participants provided informed written consent. Participants self-reported as being right-foot dominant, being physically active (engaging in at least 30 min of exercise 2× per week), and having normal or corrected-to-normal vision. Participants had no history of major musculoskeletal, neurological, or cardiovascular conditions. Individuals were excluded if they had prior experience with a split-belt treadmill, to ensure the novelty of the gait task, or were left-footed, to avoid effects of footedness on brain laterality.

### EMG setup

Neck electromyography (EMG) was recorded from the left and right sternocleidomastoid, splenius capitis, and trapezius to isolate and attenuate muscle activity within the EEG signal (see below, EEG analysis). The neck was shaved (if necessary), lightly exfoliated, and cleaned with an alcohol wipe to reduce impedance and improve signal quality. We then placed eight adhesive-fixed flat-type electrodes (*F*s = 512 Hz; Biosemi) around the neck and secured them with cover roll. The neck EMG used the same ground and reference as the EEG data and was later re-referenced.

### Dual-layer EEG setup

A custom dual-electrode system facilitated high-density EEG recordings, comprising 128 pin-type scalp electrodes and 128 flat-type “noise” electrodes (BioSemi ActiveTwo system, RRID:SCR_023671, *F*s = 512 Hz). For each scalp electrode, there is a partner “noise” electrode that is mechanically coupled but electrically isolated from the scalp electrode. This dual-electrode system exhibited a twofold enhancement in signal-to-noise ratio in a phantom head simulation experiment (Nordin et al., 2018). EMG and noise channel data aided in EEG preprocessing, which is detailed in the EEG analysis section.

Multiple steps were taken before EEG data collections to ensure high-fidelity neural signals could be recorded in a mobile setting. We asked participants to refrain from applying hair products prior to the experiment. The EEG electrode cap was placed in accordance with the international 10–20 system, and a temporary line was drawn on the participant's forehead to monitor any shift in cap location. Electrode locations were digitized using 3D head scanners (Eva scanner, Artec 3D; structure sensor on Apple® iPad; scanner, Occipital; itSeez3D). To minimize electrode–skin impedance, a blunt-tip gel syringe was used to gently part the hair and lightly abrade the scalp before applying the gel and placing the electrodes. All scalp electrode offsets were at or below 20 mV before recording. A secondary custom conductive cap mimicking scalp conductivity (Eeonyx) was placed over noise electrodes and secured with PowerFlex to prevent shifting. By connecting the noise electrodes, this secondary cap creates a circuit that is similar to the scalp electrodes, but without the biological signals. This noise electrode circuit allows us to isolate motion artifact and electrical interference from external sources. Wires were tightly bundled with Velcro straps to minimize cable sway, which is known to introduce artifacts into EEG signals (Symeonidou et al., 2018). Participants were shown the impact of their jaw clenching, excessive eye blinking, and neck muscle tension on raw live-streamed EEG data and instructed to refrain from these behaviors.

### Behavioral data

We used a force-instrumented treadmill (Bertec Fully Instrumented Treadmill, RRID:SCR_015789) and lower body motion capture (OptiTrack) to measure kinetics and stepping kinematics during split-belt treadmill walking. Force transducers beneath each belt captured foot contact events from each limb (*F*s = 1,000 Hz). Motion capture markers were placed on the medial and lateral ankles to measure foot position. A hard-wired 0.5 Hz square wave synchronized force plate, motion capture, EEG, noise channel, and EMG data.

### Experiment Design

**Figure 1.**
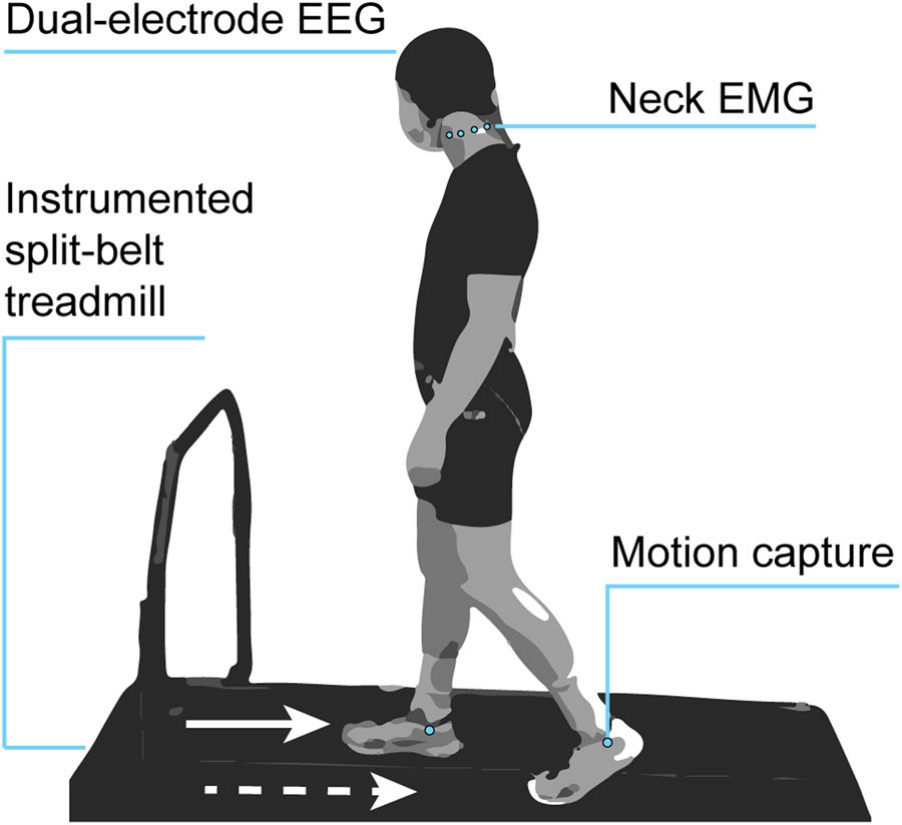
Experimental setup. Participants walked on an instrumented split-belt treadmill while equipped with a 256 dual-electrode EEG system, eight neck electromyography electrodes, and markers for motion capture on the lower body. This figure is adapted from Jacobsen and Ferris (2023).

**Figure 2.**
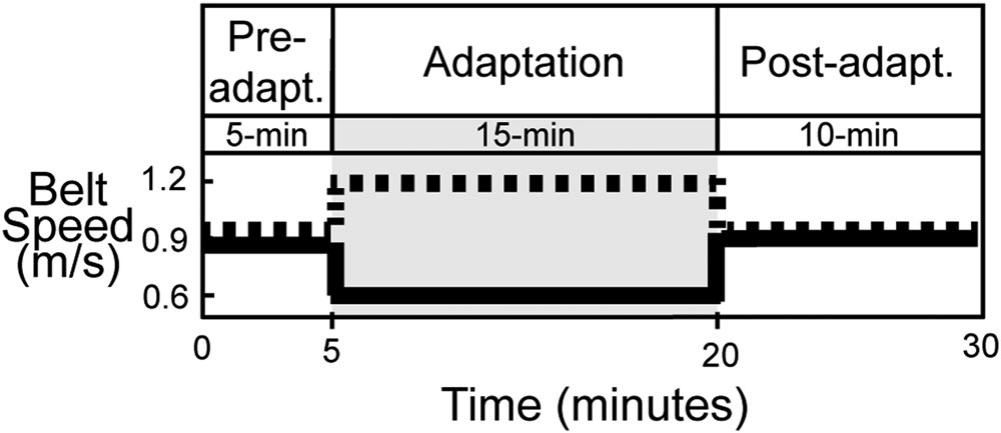
Experimental paradigm. Participants were exposed to a split-belt treadmill perturbation (2:1 speed ratio) for 15  min. During the pre- and postadaptation periods, the belts were moving at the same speed. The dashed black line indicates the speed of the right belt, and the solid black line indicates the speed of the left belt. This figure is adapted from Jacobsen and Ferris (2023).

Participants were equipped with a dual electrode EEG system, neck EMG, and lower body motion capture markers (Figure 1), while they completed a split-belt walking paradigm for 40 min. Figure 2 shows the experimental conditions, which consisted of both tied-belt and split-belt walking: 15 min of pre-adaptation (only the last 5 min are shown in Figure 2), 15 min of adaptation, and 10 min of post-adaptation. They wore comfortable walking shoes, face masks per COVID-19 safety protocols, and earplugs to reduce their ability to hear treadmill speed changes. Participants were instructed to look straight ahead, avoid looking down at their feet, and use the handrail only if necessary. They were also told to walk naturally, swinging their arms as they naturally would. They were informed when the treadmill would initially start and finally stop but were not warned about belt speed changes in between.The pre-adaptation phase involved walking at slow (v = 0.6 m/s), fast (v = 1.2 m/s), and medium (v = 0.9 m/s) speeds, which reflected the range of belt speeds experienced. For analysis, pre-adaptation refers to the medium speed condition unless otherwise indicated. During the adaptation phase, belt speeds were abruptly split to a 2:1 ratio within 1.5 seconds (vright = 1.2 m/s, vleft = 0.6 m/s, a = 0.2 m/s²) for 15 min. The right belt was always faster, corresponding to the dominant foot to avoid laterality as a confounding variable. Adaptation was followed by a 10-min post-adaptation wash-out period consisting of normal tied-belt walking (v = 0.9 m/s).

### Behavioral data analysis

Vertical ground reaction force data from the treadmill were used to identify gait events. The data were low-pass filtered (zero-phase, second-order, Butterworth filter) with a cutoff frequency of 6 Hz and downsampled to 512 Hz align with the EEG sampling rate. Foot contact and lift-off events were detected using a 10 N threshold. Time windows with abnormal gait event latencies [>4 standard deviations (SDs)] were visually inspected, and any missteps, such as stepping on the wrong force plate, were manually corrected.

We used step length asymmetry to assess behavioral performance during split-belt adaptation. Step length asymmetry is a robust measure of adaptation and can be altered by adjusting spatial and/or temporal parameters (Reisman et al., 2005). Step length was defined as the distance between the anterior–posterior positions of the ankle markers along the sagittal plane at foot contact for each leg. Any missing data points for the ankle markers were linearly interpolated. To quantify step length asymmetry, the difference in step length between the faster limb (on the faster belt) and the slower limb (on the slower belt) was normalized by the sum of the two step lengths, expressed by the following formula: step length asymmetry = [(fast step length − slow step length) / (fast step length + slow step length)] (Reisman et al., 2005). Step length asymmetry was normalized within each participant by subtracting each participant's average step length asymmetry of preadaptation walking at 0.9 m/s.

We calculated the number of strides it took to reach steady-state step length asymmetry similar to Fettrow et al. (2021) and Finley et al. (2015). Steady state was the average value calculated over the final 30 steps of adaptation. The threshold for achieving the steady state was determined when step length asymmetry remained within two SDs of the steady state for a minimum of 10 consecutive steps. Participants were sorted into two groups using a median split of the number of strides it took them to adapt, resulting in slow and fast adaptation groups.

In addition to step length asymmetry, we quantified step width and double support to evaluate behavioral changes across adaptation. Step width was measured as the mediolateral distance between ankle markers at foot contact. We used step width variability to quantify gait stability. To quantify step width variability, we calculated the SD of step width for each limb (slow belt limb and fast belt limb) across adaptation for each participant. We also calculated a double support ratio to assess symmetry. This parameter was calculated by first quantifying percent double limb support, which was the time when both feet are in contact with the treadmill, expressed as a percentage of stride time for each leg. We define slow double support as occurring at the end of the slow limb's stance (i.e., the time from right foot contact to left toe-off) and vice versa for fast double support. We then calculated the double support ratio (fast double support:slow double support; Reisman et al., 2005). For all behavioral metrics, we removed outlier steps that were greater than three SDs from the mean and excluded epochs that did not have an event sequence of a full gait cycle.

We defined subconditions of gait adaptation using stride binning from each condition: preadaptation (mean of last 30 strides), initial adaptation (mean of strides 1–10), early adaptation (mean of strides 11–40), and late adaptation (mean of last 30 strides). The same stride binning was repeated for the postadaptation period. Initial and early adaptations were differentiated because we wanted to separately examine the initial reaction period following the abrupt perturbation. Roemmich and Bastian (2015) used 5 strides for the initial adaptation, 25 strides for the early adaptation, and 30 strides for the late adaptation. We chose 10 instead of 5 strides because of the high variability of the EEG data and instability following an abrupt perturbation. The initial adaptation period is most prone to motion artifacts because this is when participants are unstable and off-balance. We wanted all remaining subconditions to have the same number of strides for consistency. This was the same stride binning used in Jacobsen and Ferris (2023).

### Volume conduction modeling

To improve EEG source localization, we created personalized head models for each participant using individual structural magnetic resonance images (T1-MRI from 3 T Philips Ingenia scanner) and the FieldTrip-SIMBIO pipeline (Vorwerk et al., 2018). Realistic head models improve the accuracy of the EEG inverse solution (Roth et al., 1993; Cuffin, 1996; Haueisen et al., 1997; Vanrumste et al., 2002; Akalin Acar and Makeig, 2013). The head models were constructed using the finite element method and coregistered with digitized electrode locations using fiducial markers. Each model had five compartment layers with specific tissue conductivity values: skin = 0.43 S/m, skull = 0.01 S/m, cerebral spinal fluid = 1.79 S/m, gray matter = 0.33 S/m, and white matter = 0.14 S/m (Baumann et al., 1997; Haueisen et al., 1997; Hoekema et al., 2003; Vorwerk et al., 2019).

### EEG analysis

The EEG processing pipeline was executed using MATLAB 2022a (MathWorks), EEGLAB (v2022.0, RRID:SCR_007292; Delorme and Makeig, 2004), additional toolboxes mentioned later, and customized scripts. Using a combination of standard and innovative preprocessing steps, we cleaned the EEG data. Data were high-pass filtered at 1 Hz (zero-phase, second-order, Butterworth) to mitigate slow drift. Scalp, muscle, and noise channels were individually re-referenced using the average across channels after the removal of bad channels (>3 SDs). On average 4 ± 3.8 (mean ± SD) channels were removed based on this criterion. To diminish motion and muscle artifacts, we used the iCanClean algorithm (Downey and Ferris, 2023), employing canonical correlation analysis for effective noise reduction. We used three cleaning cycles to attenuate stationary noise, nonstationary noise, and muscle artifacts. In the first cycle, we removed components of scalp and EMG channels that correlated with components from noise channels in an infinite window (*R*^2^ = 0.05). The second cycle similarly removed subspaces of noise components but within a 2 s window (*R*^2^ = 0.95). In the last cycle, we removed components of scalp channels that correlated with components from the EMG channels in a 2 s window (*R*^2^ = 0.95). Subsequently, cleanline() (Mullen, 2012) eliminated 60 Hz line noise. Bad channels were excluded based on statistical criteria: probability (absolute threshold = 5), standard deviation (SD > 500), and kurtosis (absolute threshold = 5; Gwin et al., 2011). The clean_artifacts() tool clean_rawdata() (v 2.91, default parameters) was used to reject noisy time windows and channels (Kothe et al., 2019). On average, 119 channels (±6 SDs) and 95% of frames were retained in each dataset. The EEG data were re-referenced to the common average, maintaining full rank. EEG, ground reaction force, and motion capture data were consolidated into a unified dataset using a hard-wired synchronization waveform (0.5 Hz).

Subsequently, we moved to source-level analysis. The preprocessed EEG data, averaging 50 min per person, was decomposed using adaptive mixture independent component analysis (Palmer et al., 2012). Component data were downsampled to 256 Hz, and an equivalent dipole model for each independent component was computed using custom head models and FieldTrip software (v20210614, RRID:SCR_004849; Oostenveld et al., 2011). Following source localization, artifact components were excluded based on criteria including positive power spectral density [linear slope (2–40 Hz) > 0], high residual variance (>15%), localization outside the skull, low probability (<50%) of being a brain component per iclabel() (Pion-Tonachini et al., 2019), and high cross-frequency power–power coupling (quantified with PowPowCat; Thammasan and Miyakoshi, 2020) in specific frequency bands characteristic of eye and muscle components [correlation coefficient > 0.3 in low (<8 Hz)- and high (>30 Hz)-frequency windows].

An average of 21 (±7 SDs) components per participant was used for group clustering via *k*-means. All participants were used in group clustering, regardless of adaptation performance. Clustering relied on two equally weighted spatial features, scalp topography and dipole location, excluding time–frequency features to avoid potential false-positive rate inflation (Kriegeskorte et al., 2009). The optimal cluster number was estimated using evalclusters() and the average of results from the Calinski–Harabasz, silhouette, and Davies–Bouldin methods. When multiple components per participant were present within a cluster, the components explaining the greatest variance (i.e., the lowest independent component order number) were chosen to prevent artificial sample size inflation. Each dipole cluster was then replotted with one of two colors after participants were sorted in to slow and fast adaptation groups.

This study aimed to enhance our understanding of the neural correlates of gait adaptation, with a specific focus on identifying distinct electrocortical signatures associated with slow and fast adapters. Therefore, our analysis focused on independent component clusters within brain regions known for sensorimotor adaptation: the sensorimotor cortex, anterior cingulate cortex, posterior parietal cortex, and visual cortex. We performed time–frequency analyses for each dipole cluster by adaptation group (slow vs fast). We computed single-trial spectrograms in decibel log scale using a fast Fourier transform algorithm via std_precomp(), which calls newtimef(). We generated event-related spectral perturbation plots and standardized them through single-trial baseline removal (averaging log power across time within one gait cycle) using the following parameters in std_precomp(): Morlet wavelet cycles, (3, 0.8); pad ratio, 2; basenorm, “on”; baseline, NaN (manual baseline subtraction applied later); and triabase, “full”. This single-trial baseline normalization method has been demonstrated to be less susceptible to noisy trials (Grandchamp et al., 2011). Additionally, we subtracted the mean event-related spectral power during preadaptation from all event-related spectral perturbation plots. Event-related spectral perturbations were linearly time-warped using EEGLAB's timewarp() to the group median gait cycle length using foot lift-off and contact events. The group mean event-related spectral perturbation plots were constructed by averaging the event-related spectral perturbations of all independent components within an adaptation group cluster.

### Statistical analysis

We conducted statistical analyses to evaluate gait-related spectral power during different stages of adaptation and to examine differences between slow and fast adapters. We quantified gait-related spectral power during initial adaptation, early adaptation, and late adaptation with respect to preadaptation (0.9 m/s) for both groups. We also quantified the difference in gait-related spectral power between slow and fast adapters by subtracting the slow group's gait-related spectral plot from the fast group's gait-related spectral plot (ERSPfast adapters − ERSPslow adapters = ERSPgroup difference). As an exploratory analysis, we used cluster-based permutation tests to evaluate the effect of condition (adaptation subcondition vs preadaptation) and group (fast vs slow) on event-related spectral power (2–50 Hz). We conducted statistical analyses using cluster-based permutation tests calculated with FieldTrip (Oostenveld et al., 2011) functions within EEGLAB (Delorme and Makeig, 2004), which uses the Monte Carlo method to estimate permutation *p*-values. Cluster-based permutation testing is a robust nonparametric statistical approach that corrects for multiple comparisons and reduces the potential for false negatives in high-dimensional EEG data (Maris and Oostenveld, 2007). The multiple-comparisons problem was solved using the maximum of the cluster-level test statistic [std_stat() parameters: fieldtripmcorrect, “cluster”; fieldtripmethod, “montecarlo”; remaining ft_statistics_montecarlo() parameters set to default values]. *P*-values were approximated by calculating Monte Carlo significance probability, which addresses multiple comparisons. All cluster-based permutation tests were performed with a significance threshold of 0.05 and involved 10,000 permutations. Please note that cluster-based permutation tests do not establish significance of effect latency or frequency (Sassenhagen and Draschkow, 2019). Hence, observations of spectral power changes within specific frequency bands (e.g., alpha), which is narrower than the tested frequency range (2–50 Hz), at particular timepoints during the gait cycle are purely descriptive.

Because the cluster-based permutation tests indicated that the posterior parietal, bilateral sensorimotor, and right visual cortices displayed an effect of group on gait-related spectral power, we performed an additional analysis to examine how spectral power changed over the course of adaptation. We focused this exploratory analysis only on frequency bands where we descriptively observed prominent spectral fluctuations: alpha (8–12 Hz) for the posterior parietal and right visual cortices and theta (4–7 Hz) for the bilateral sensorimotor cortex. We computed group-average spectral power across the frequency band of interest for each gait cycle. We determined the number of strides to examine based off the participant with the fewest number of strides from adaptation. This analysis allowed us to observe how group spectral power changed on a stride-by-stride basis throughout the entire adaptation period, rather than just during subconditions of adaptation.

To statistically evaluate behavioral differences, we compared step length asymmetry, step width, step width variability, and double support ratio across groups. Kolmogorov–Smirnov tests indicated the behavioral data were not normally distributed. We used cluster-based permutation to test for group step length asymmetry, step width, and double support ratio differences over the course of adaptation (*α* = 0.05). We selected this method because it addresses multiple comparisons (Maris and Oostenveld, 2007), facilitates the analysis of non-normally distributed data across all strides of adaptation without the need for stride binning, and ensures consistency with EEG statistical testing. A Wilcoxon rank-sum test was used to compare group differences in step width variability. We used the minimum number of strides from adaptation across all participants so that the number of strides would be consistent.

## Results

### Demographics

Table 1 summarizes the participant demographics. Prior to any data processing, we discarded datasets from three participants due to issues with EEG data recording (*n* = 2) or protocol errors (*n* = 1). One additional participant was removed as an outlier because they showed no change in step length asymmetry, as determined by the slope of the linear fit. The group analysis included data from the remaining 29 participants, which included 14 females and 15 males. The fast group (*n* = 15) had a mean age of 22.3 ± 2.6 years [range = (19, 26)] and body mass index of 23.70 ± 4.11 (mean ± SD). The slow group (*n* = 14) was slightly older, with a mean age of 23.2 ± 2.7 years [range = (21, 29)] and a body mass index of 23.80 ± 3.84 (mean ± SD). Descriptively, there were no large differences in age, body mass index, or sex distribution between groups.

**Table 1.** Participant demographics

Sample characteristics	Fast group				Slow group			
	*n*	%	Mean	SD	*n*	%	Mean	SD
Gender								
Female	6	40			8	57		
Male	9	60			6	43		
Age (years)			22.38	2.6			23.2	2.7
Body mass index			23.70	4.11 3.85			23.80	3.84

*N* = 29, SD, standard deviation.

### Behavioral analysis

**Figure 3.**
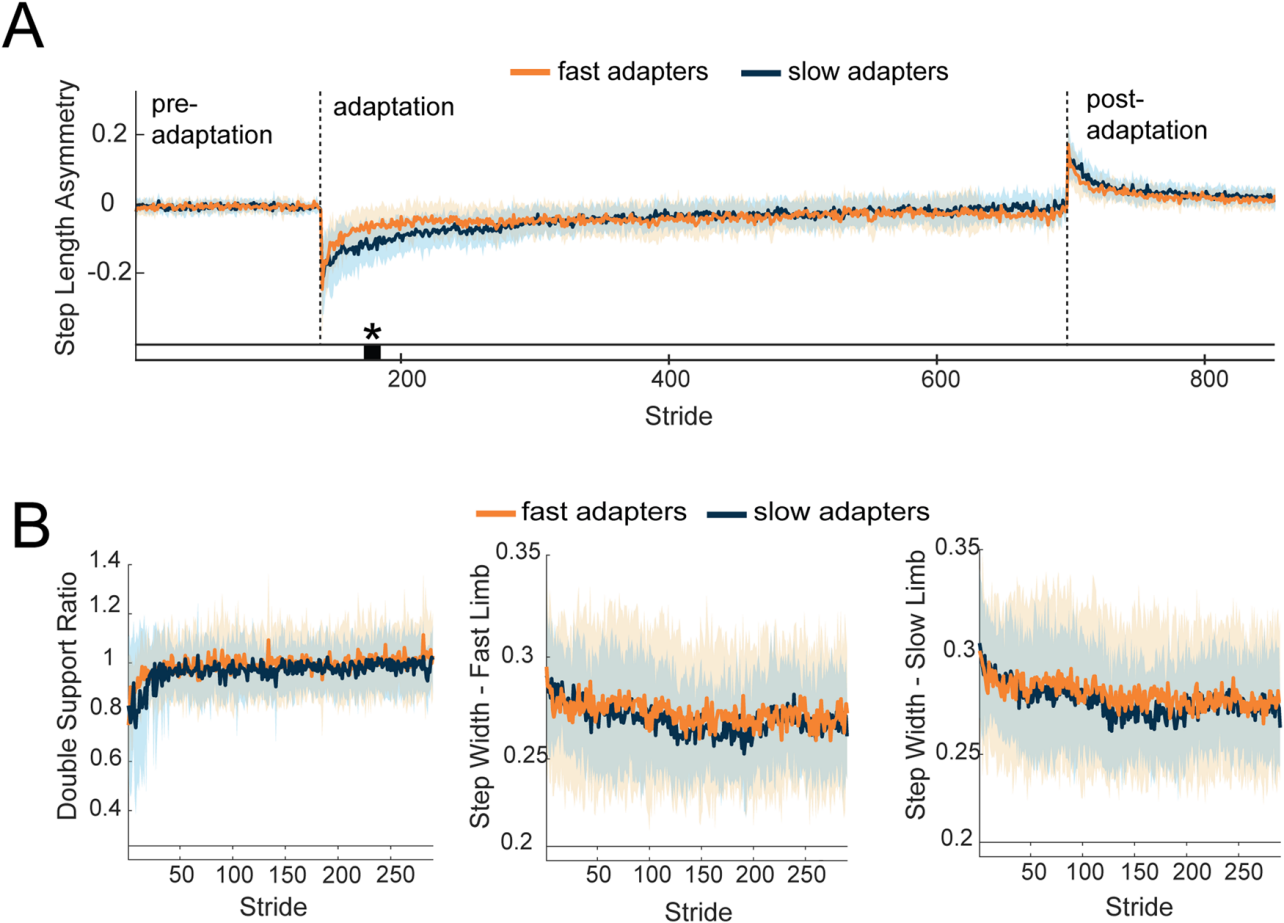
***A***, Mean ± SD adaptation curves across participants within the fast (orange; *n* = 15) and slow (blue; *n* = 14) adaptation groups. The black bar and asterisk (*) indicate a significant difference between groups (*p* < 0.05). ***B***, Mean ± SD of additional behavioral metrics from the fast (orange) and slow (blue) adaptation groups during the adaptation period only. Data are truncated in length to match the participant who took the fewest strides during adaptation. From left to right, the behavioral metrics shown are double support ratio, step width of the fast limb (on the right belt), and step width of the slow limb (on the left belt). The empty bar below each plot indicates there were no statistical differences found between groups following cluster-based permutation tests.

We assessed the effect of the split-belt treadmill on behavioral measures during adaptation, including step length asymmetry, step width, and double support ratio (Fig. 3). Step length asymmetry exhibited differences between slow and fast adapters, which were expected given that this metric was used to split participants into groups. The fast group took an average of 60 [±36 SDs, range = (6, 116)] strides to reach steady-state step length asymmetry, and the slow group took 238 [±106 SDs, range = (123, 541)]. Both fast and slow adapters increased step length asymmetry during split-belt adaptation and demonstrated aftereffects during postadaptation (Fig. 3*A*). A cluster-based permutation test indicated there was an effect of group on step length asymmetry (*p* = 0.02), which confirms that the “fast” group indeed adapted faster than the “slow” group (Fig. 3*A*). Despite having different adaptation rates during early adaptation, the deadaptation rates between the groups were similar. There were no differences in step width, step width variability, or double support ratio between groups during adaptation (cluster-based permutation, *p* > 0.05; Fig. 3*B*). Wilcoxon signed-rank tests indicated there was not a significant difference between slow and fast adapters’ step width variability during adaptation (slow belt leg, *p* = 0.56; fast belt leg, *p* = 1.0).

### Group electrocortical activity during split-belt adaptation

**Figure 4.**
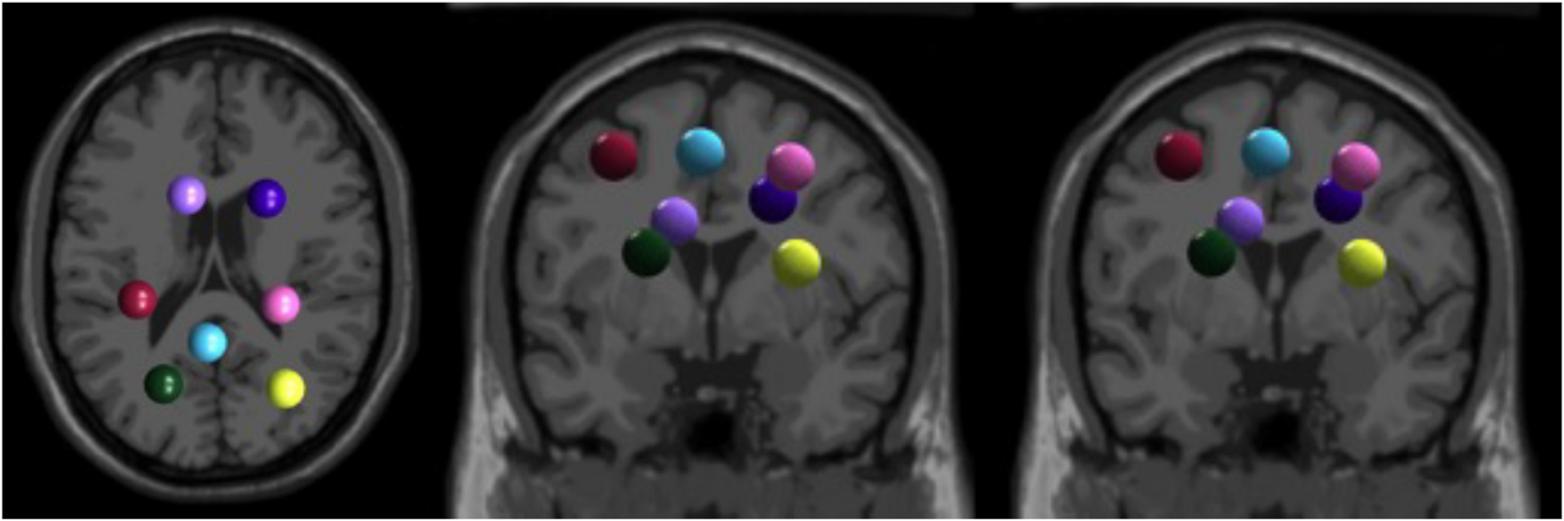
Independent component cluster centroids. Independent component cluster centroids (large spheres) from all participants are plotted on a template brain image from the Montreal Neurological Institute and viewed in the axial (left), coronal (middle), and sagittal (right) planes. These cluster centroids give approximate locations of the electrophysiological sources: the left anterior cingulate (light purple), right anterior cingulate (dark purple), and left sensorimotor (maroon), right sensorimotor (pink), posterior parietal (blue), left visual (green), and right visual cortices (lime green/yellow).

We identified multiple neural source clusters that contained independent components from at least half of the participants (*n* ≥ 15). The independent component clusters that we focused our analysis on were the posterior parietal cortex and bilateral sensorimotor, anterior cingulate, and visual cortices. The centroids of these clusters are plotted in Figure 4 on a template brain image from the Montreal Neurological Institute.

**Figure 5.**
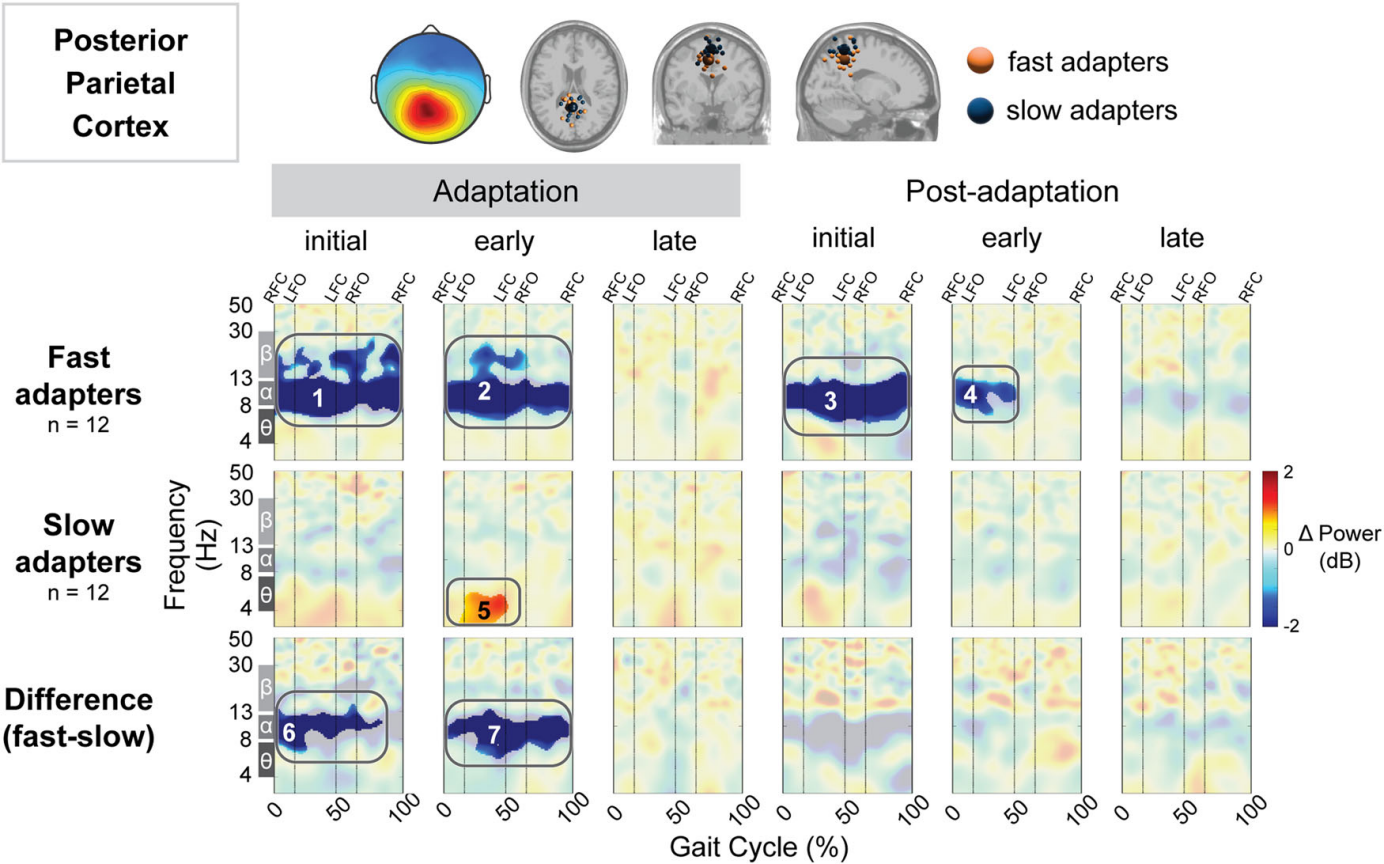
Group differences in gait-related spectral differences for an independent component cluster located near the posterior parietal cortex. Top: Mean scalp projection and equivalent dipole locations of the cluster-independent components by group (orange, fast adapters; blue, slow adapters) in the axial, coronal, and sagittal plane (from left to right). Bottom: Mean event-related spectral perturbation plots with respect to preadaptation for each group and the difference between groups. We assessed the significance of condition (adaptation subcondition vs preadaptation) for each group and the significance of group (fast vs slow adapters) using cluster-based permutation tests (nfast = 12, nslow = 12). Nonsignificant differences have a transparent white mask. Vertical dashed lines indicate gait events: right foot contact (RFC), left foot-off (LFO), left foot contact (LFC), and right foot-off (RFO).

**Figure 6.**
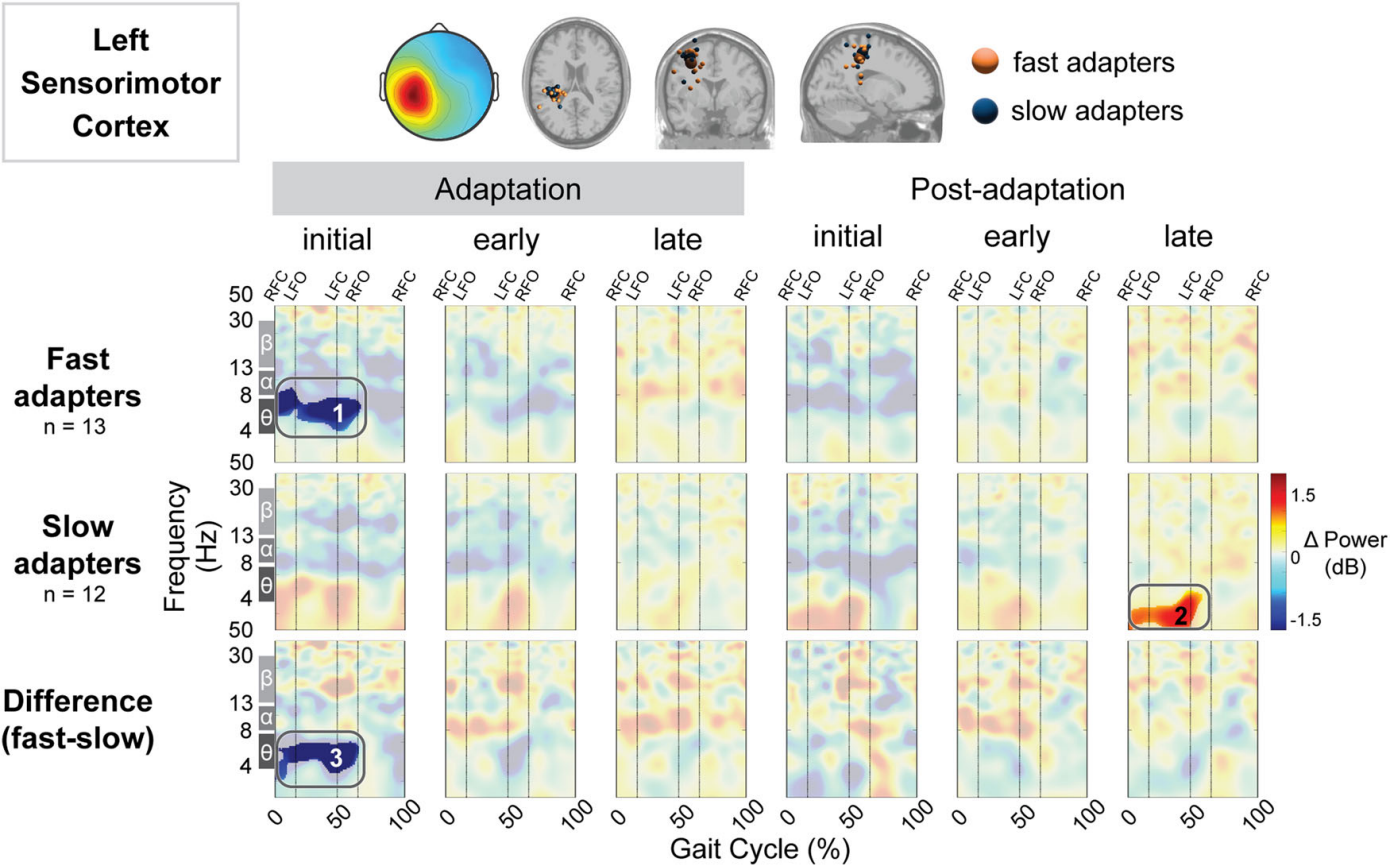
Group differences in gait-related spectral differences for an independent component cluster located near the left sensorimotor cortex. Top: Mean scalp projection and equivalent dipole locations of the cluster-independent components by group (orange, fast adapters; blue, slow adapters) in the axial, coronal, and sagittal plane (from left to right). Bottom: Mean event-related spectral perturbation plots with respect to preadaptation for each group and the difference between groups. We assessed the significance of condition (adaptation subcondition vs preadaptation) for each group and the significance of group (fast vs slow adapters) using cluster-based permutation tests (nfast = 13, nslow = 13). Nonsignificant differences have a transparent white mask. Vertical dashed lines indicate gait events: right foot contact (RFC), left foot-off (LFO), left foot contact (LFC), and right foot-off (RFO).

**Figure 7.**
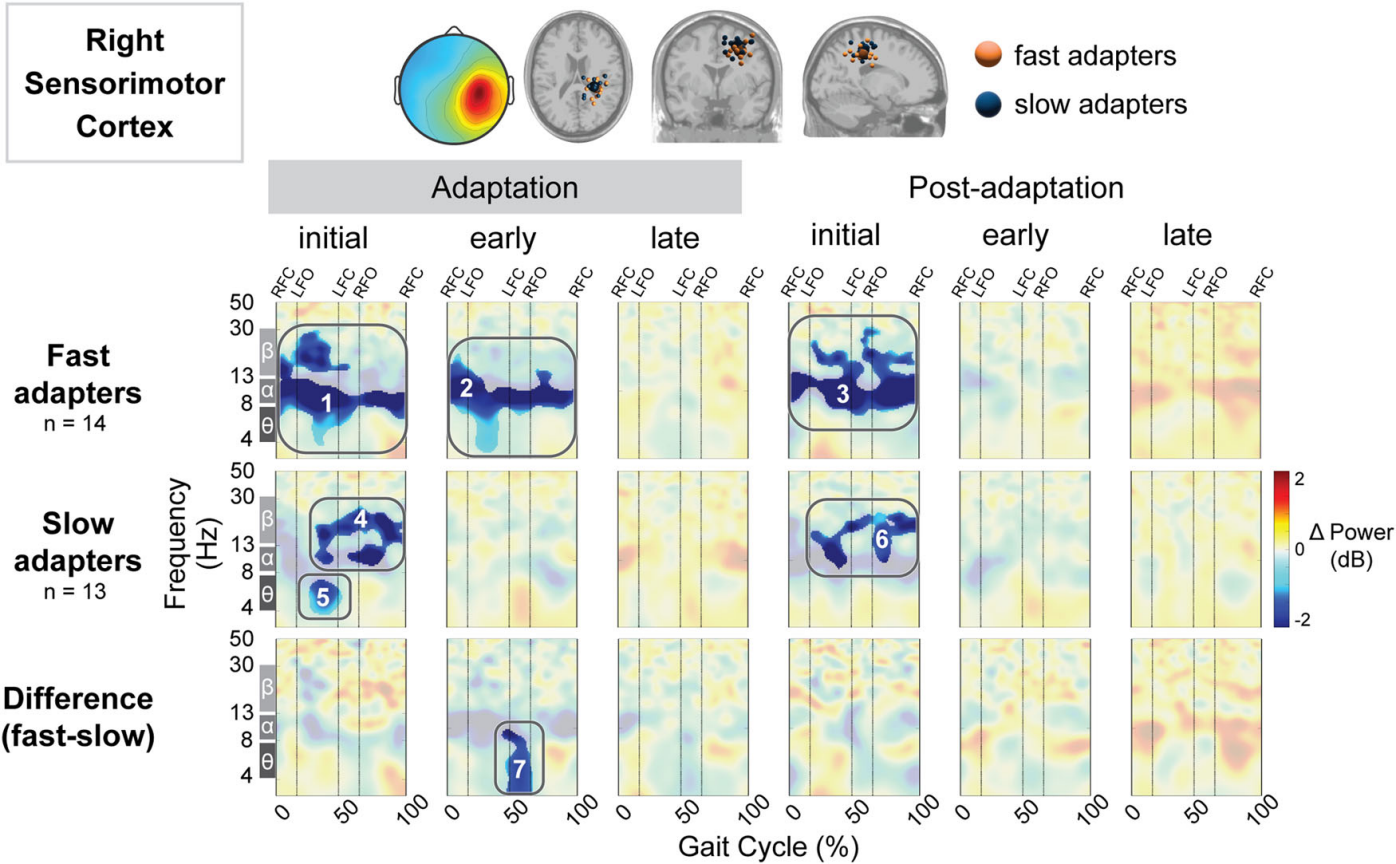
Group differences in gait-related spectral differences for an independent component cluster located near the right sensorimotor cortex. Top: Mean scalp projection and equivalent dipole locations of the cluster-independent components by group (orange, fast adapters; blue, slow adapters) in the axial, coronal, and sagittal plane (from left to right). Bottom: Mean event-related spectral perturbation plots with respect to preadaptation for each group and the difference between groups. We assessed the significance of condition (adaptation subcondition vs preadaptation) for each group and the significance of group (fast vs slow adapters) using cluster-based permutation tests (nfast = 12, nslow = 12). Nonsignificant differences have a transparent white mask. Vertical dashed lines indicate gait events: right foot contact (RFC), left foot-off (LFO), left foot contact (LFC), and right foot-off (RFO).

**Figure 8.**
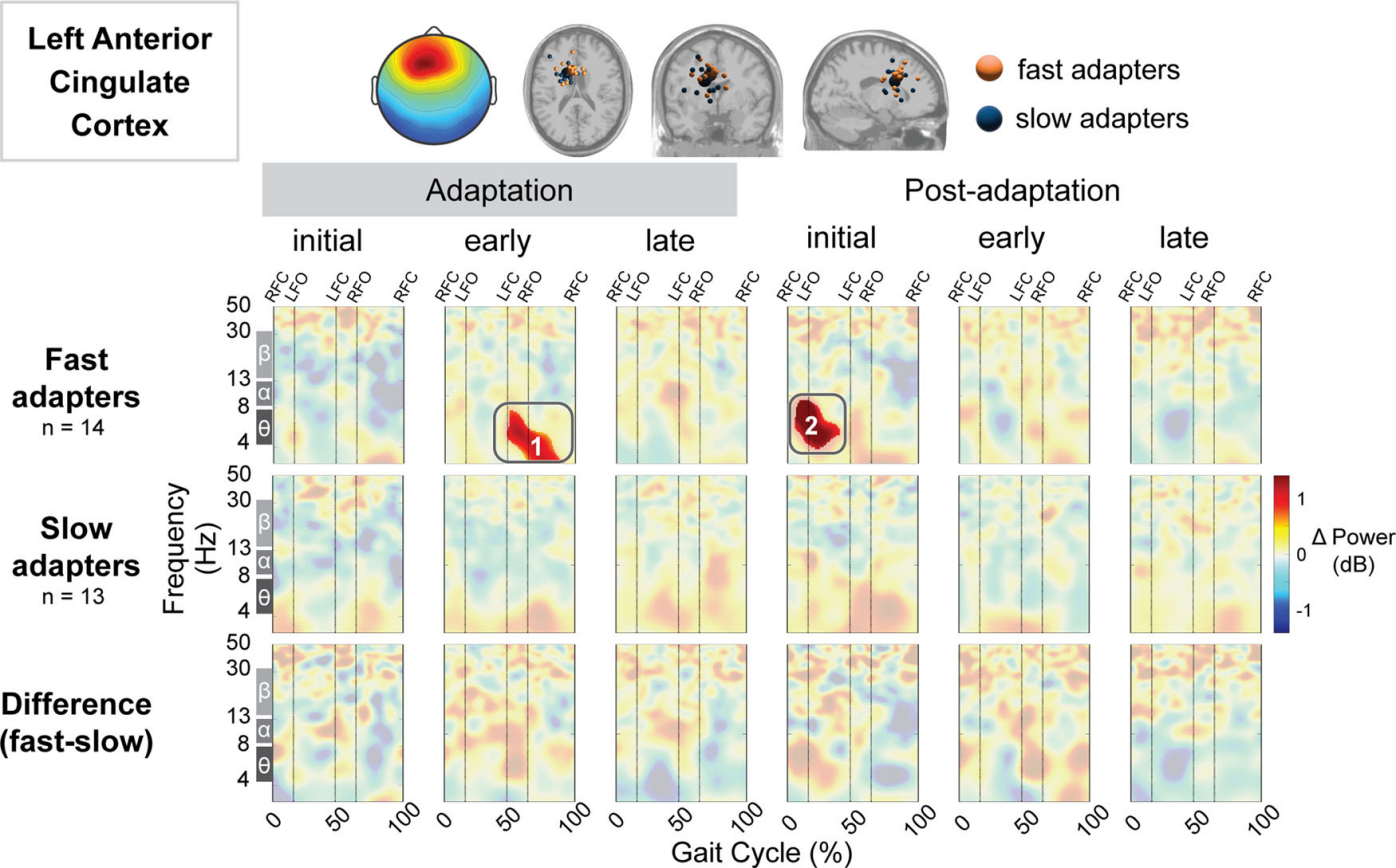
Group differences in gait-related spectral differences for an independent component cluster located near the left anterior cingulate cortex. Top: Mean scalp projection and equivalent dipole locations of the cluster-independent components by group (orange, fast adapters; blue, slow adapters) in the axial, coronal, and sagittal plane (from left to right). Bottom: Mean event-related spectral perturbation plots with respect to preadaptation for each group and the difference between groups. We assessed the significance of condition (adaptation subcondition vs preadaptation) for each group and the significance of group (fast vs slow adapters) using cluster-based permutation tests (nfast = 13, nslow = 13). Nonsignificant differences have a transparent white mask. Vertical dashed lines indicate gait events: right foot contact (RFC), left foot-off (LFO), left foot contact (LFC), and right foot-off (RFO).

**Figure 9.**
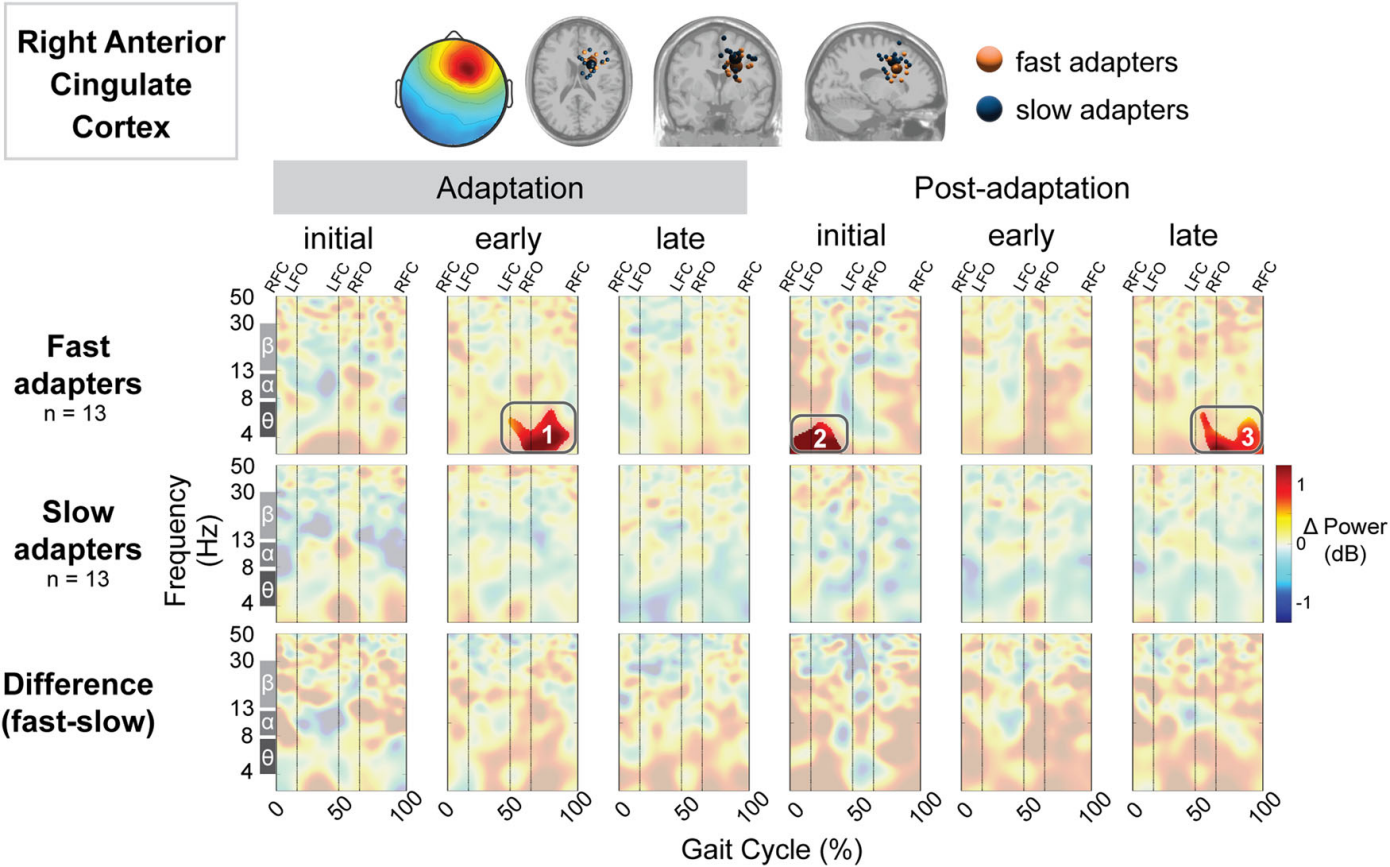
Group differences in gait-related spectral differences for an independent component cluster located near the right anterior cingulate cortex. Top: Mean scalp projection and equivalent dipole locations of the cluster-independent components by group (orange, fast adapters; blue, slow adapters) in the axial, coronal, and sagittal plane (from left to right). Bottom: Mean event-related spectral perturbation plots with respect to preadaptation for each group and the difference between groups. We assessed the significance of condition (adaptation subcondition vs preadaptation) for each group and the significance of group (fast vs slow adapters) using cluster-based permutation tests (nfast = 13, nslow = 13). Nonsignificant differences have a transparent white mask. Vertical dashed lines indicate gait events: right foot contact (RFC), left foot-off (LFO), left foot contact (LFC), and right foot-off (RFO).

**Figure 10. EN-NWR-0515-23F10:**
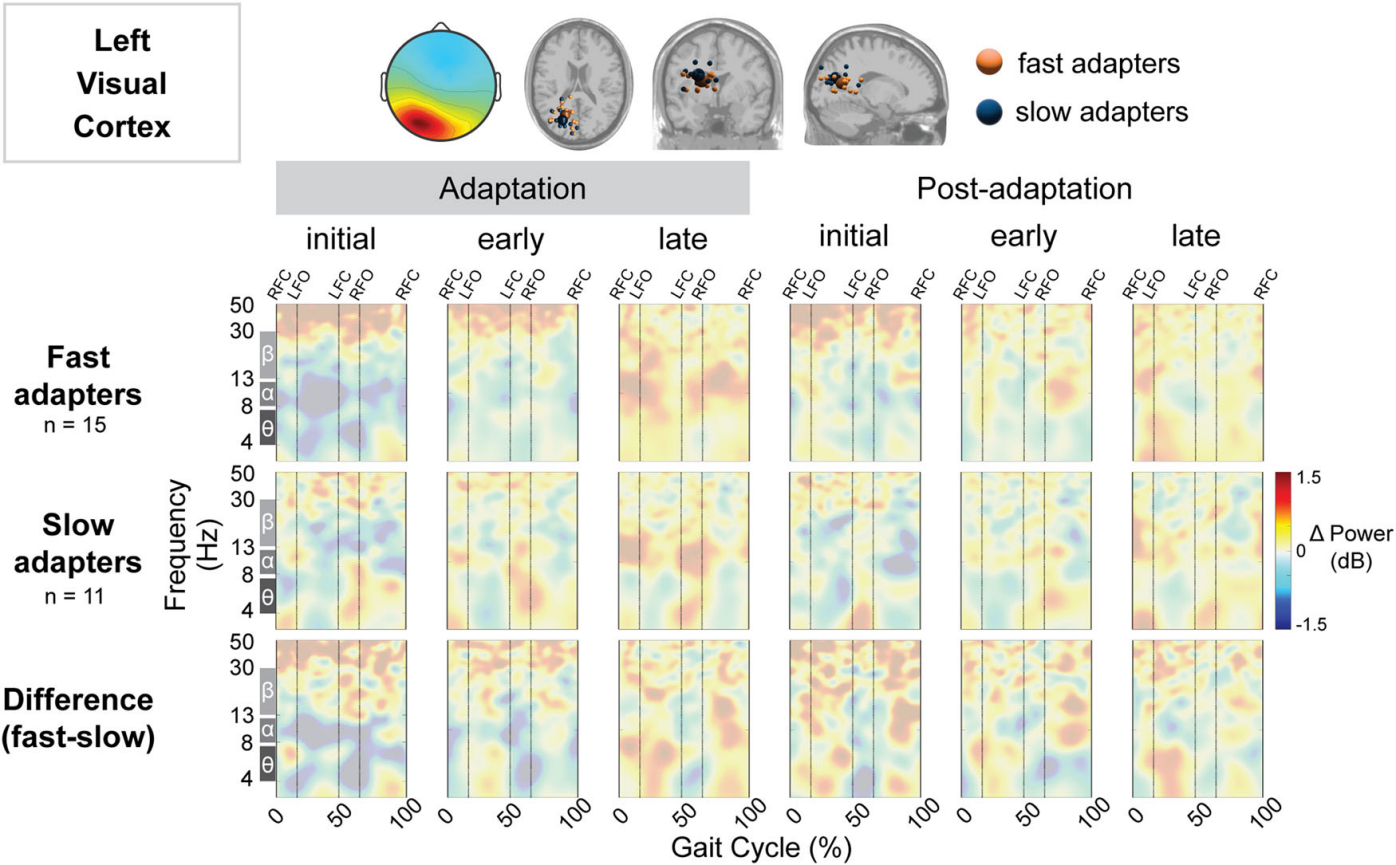
Group differences in gait-related spectral differences for an independent component cluster located near the left visual cortex. Top: Mean scalp projection and equivalent dipole locations of the cluster-independent components by group (orange, fast adapters; blue, slow adapters) in the axial, coronal, and sagittal plane (from left to right). Bottom: Mean event-related spectral perturbation plots with respect to preadaptation for each group and the difference between groups. We assessed the significance of condition (adaptation subcondition vs preadaptation) for each group and the significance of group (fast vs slow adapters) using cluster-based permutation tests (nfast = 14, nslow = 11). Nonsignificant differences have a transparent white mask. Vertical dashed lines indicate gait events: right foot contact (RFC), left foot-off (LFO), left foot contact (LFC), and right foot-off (RFO).

**Figure 11.**
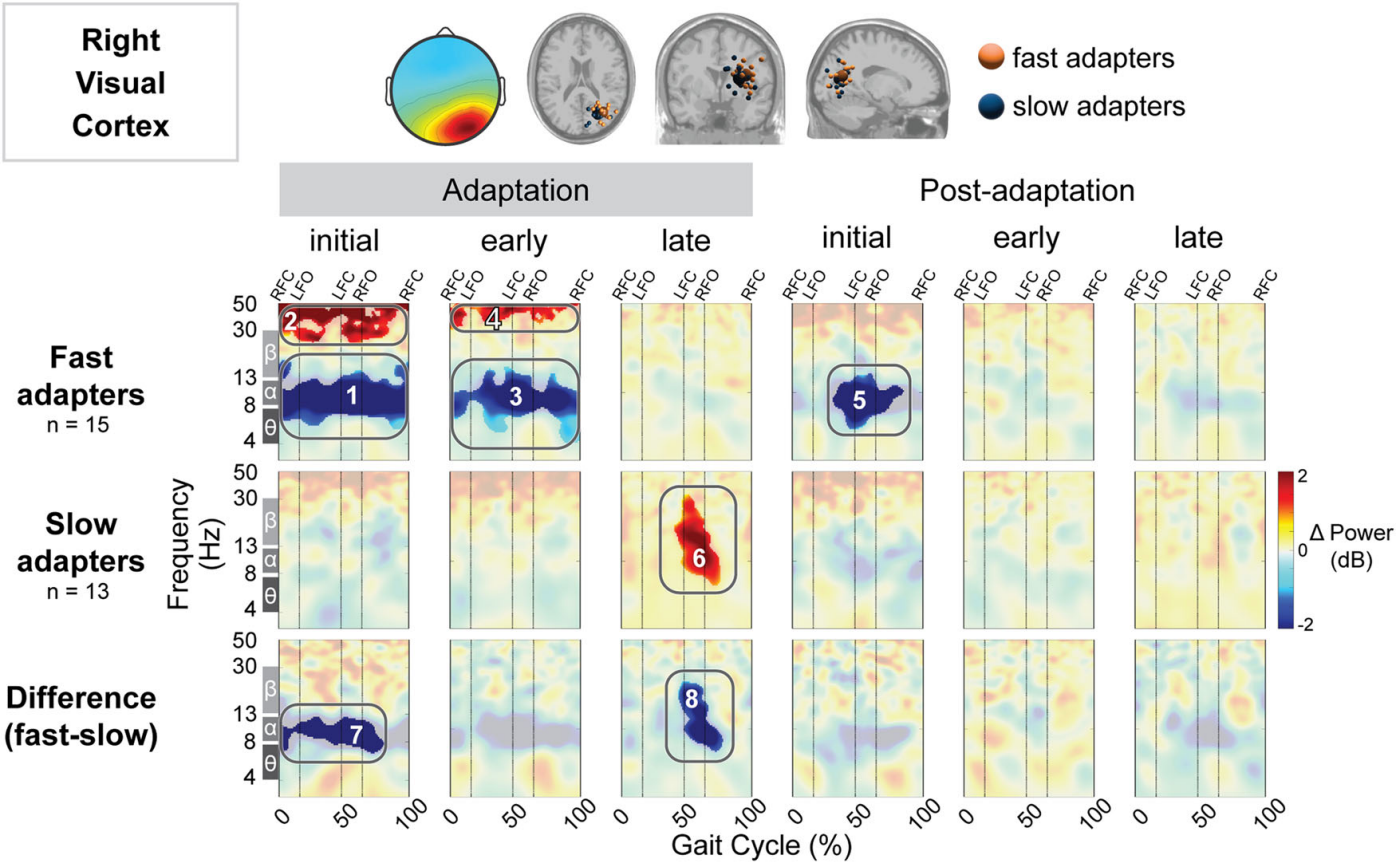
Group differences in gait-related spectral differences for an independent component cluster located near the right visual cortex. Top: Mean scalp projection and equivalent dipole locations of the cluster-independent components by group (orange, fast adapters; blue, slow adapters) in the axial, coronal, and sagittal plane (from left to right). Bottom: Mean event-related spectral perturbation plots with respect to preadaptation for each group and the difference between groups. We assessed the significance of condition (adaptation subcondition vs preadaptation) for each group and the significance of group (fast vs slow adapters) using cluster-based permutation tests (nfast = 14, nslow = 13). Nonsignificant differences have a transparent white mask. Vertical dashed lines indicate gait events: right foot contact (RFC), left foot-off (LFO), left foot contact (LFC), and right foot-off (RFO).

We quantified gait-related spectral power in multiple cortical regions (Figs. 5–11). We computed the group mean gait-related spectral power across stages of adaptation with respect to preadaptation and the difference between groups (Fig. 5). Red indicates increased spectral power (neural synchronization) and blue indicates decreased spectral power (neural desynchronization). Above these spectral plots are the scalp topography and dipole locations for each cluster (Fig. 5, top row). Cluster-based permutation tests revealed an effect of group (slow vs fast) on gait-related spectral power in four brain regions: posterior parietal (Fig. 5), left sensorimotor (Fig. 6), right sensorimotor (Fig. 7), and right visual (Fig. 11) cortices. All of the significant group differences occurred during one or more of the adaptation conditions; there were no group differences in spectral power during the postadaptation period in any of the cortical clusters analyzed. Table 2 includes all statistical results, including effect sizes, from cluster-based permutation tests on the effect of condition and group on gait-related spectral power.

**Table 2.** Significant cluster-based permutation test results on the effect of condition and group on power spectral density (2–50 Hz)

Cortical cluster label	Group	Conditions	Circled region no.	Δ power w.r.t. preadapt.	*p*-value	df	Max effect size	95% confidence interval
Right sensorimotor	Fast	Initial postadapt. versus preadapt.	3	−	<0.001	14	1.72	[0.93, 2.60]
Right sensorimotor	Slow	Initial postadapt. versus preadapt.	6	−	0.008	13	1.23	[0.33, 2.11]
Right sensorimotor	Fast	Early adapt. versus preadapt.	2	−	0.003	14	0.68	[−0.26, 1.45]
Right sensorimotor	Fast	Initial adapt. versus preadapt.	1	−	0.000	14	0.48	[−0.26, 1.25]
Right sensorimotor	Slow	Initial adapt. versus preadapt.	4	−	0.004	13	1.27	[0.20, 2.29]
Right sensorimotor	Slow	Initial adapt. versus preadapt.	5	−	0.024	13	1.65	[0.81, 2.79]
Right sensorimotor	Both	Early adapt. group difference	7	−	0.024	(14,13)	−0.25	[−1.11, 0.54]
Left anterior cingulate	Fast	Initial postadapt. versus preadapt.	2	+	0.007	14	1.07	[0.26, 1.86]
Left anterior cingulate	Fast	Early adapt. versus preadapt.	1	+	0.004	14	0.22	[−0.54, 1.04]
Posterior parietal	Fast	Early postadapt. versus preadapt.	4	−	0.018	12	−0.09	[−1.01, 0.81]
Posterior parietal	Fast	Initial postadapt. versus preadapt.	3	−	<0.001	12	0.70	[−0.11, 1.52]
Posterior parietal	Fast	Early adapt. versus preadapt.	2	−	0.001	12	0.11	[−0.70, 0.94]
Posterior parietal	Slow	Early adapt. versus preadapt.	5	+	0.011	12	0.45	[−0.28, 1.27]
Posterior parietal	Fast	Initial adapt. versus preadapt.	1	−	<0.0001	12	1.88	[0.82, 3.02]
Posterior parietal	Both	Initial adapt. group difference	6	−	0.010	(12,12)	1.12	[0.34, 2.00]
Posterior parietal	Both	Early adapt. group difference	7	−	0.007	(12,12)	1.33	[0.60, 2.13]
Right visual	Fast	Initial postadapt. versus preadapt.	5	−	0.023	15	0.79	[0.01, 1.49]
Right visual	Fast	Early adapt. versus preadapt.	3	−	0.001	15	0.18	[−0.58, 0.97]
Right visual	Fast	Early adapt. versus preadapt.	4	+	0.021	15	−0.03	[−0.75, 0.74]
Right visual	Slow	Late adapt. versus preadapt.	6	+	0.007	13	0.00	[−0.86, 0.77]
Right visual	Fast	Initial adapt. versus preadapt.	2	+	0.001	15	1.22	[0.38, 1.99]
Right visual	Fast	Initial adapt. versus preadapt.	1	−	0.006	15	0.42	[−0.42, 1.04]
Right visual	Both	Initial adapt. group difference	7	−	0.009	(15,13)	1.08	[0.43, 1.82]
Right visual	Both	Late adapt. group difference	8	−	0.011	(15,13)	−0.56	[−1.25, 0.28]
Right anterior cingulate	Fast	Late postadapt. versus preadapt.	3	+	0.008	13	−0.81	[−1.60, −0.05]
Right anterior cingulate	Fast	Initial postadapt. versus preadapt.	2	+	0.015	13	0.74	[−0.13, 1.33]
Right anterior cingulate	Fast	Early adapt. versus preadapt.	1	+	0.009	13	−0.39	[−1.17, 0.45]
Left sensorimotor	Slow	Late postadapt. versus preadapt.	2	+	0.004	12	−0.73	[−1.56, 0.09]
Left sensorimotor	Fast	Initial adapt. versus preadapt.	1	−	0.008	13	0.74	[−0.05, 1.37]
Left sensorimotor	Both	Initial adapt. group difference	3	−	0.007	(13,12)	0.29	[−0.73, 1.13]

Significance threshold set at *a* = 0.05. Maximum effect size measure by Cohen's *d*. w.r.t, with respect to; df, degrees of freedom.

There were significant spectral fluctuations in the posterior parietal cortex both within and between groups during multiple stages of adaptation and postadaptation. With respect to preadaptation, fast adapters demonstrated decreased spectral power in the posterior parietal cortex during initial (Region 1; Fig. 5) and early adaptation (Region 2; Fig. 5), whereas slow adapters exhibited increased spectral power during early adaptation (Region 5; Fig. 5). Fast adapters also displayed decreased spectral power during initial and early postadaptation (Region 3 and 4, respectively; Fig. 5). When comparing the two groups, fast adapters exhibited decreased spectral power in the posterior parietal cortex with respect to slow adapters during initial (Region 6; Fig. 5) and early (Region 7; Fig. 5) adaptation. Descriptively, fast adapters exhibited decreased alpha power near the posterior parietal cortex compared with slow adapters during the initial and early stages of adaptation. In the fast adaptation group, we observed this enhanced alpha suppression across the entire gait cycle. By late adaptation, there were no significant differences between the groups.

We observed a significant effect of condition on spectral power in both the left and right sensorimotor cortices. Specifically, the fast group exhibited lower spectral power compared with both preadaptation (Region 2; Fig. 6) and the slow group during initial adaptation (Region 3; Fig. 6) in the left sensorimotor cortex. Descriptively, this reduction in spectral power was most prominent in the theta band. Conversely, the slow group showed increased spectral power only during late postadaptation (with respect to preadaptation; Region 2; Fig. 6) near the left sensorimotor cortex. Near the right sensorimotor cortex, both groups exhibited decreased spectral power during initial adaptation and postadaptation. Descriptively, fast adapters displayed desynchronization primarily within the theta, alpha, and beta bands during initial adaptation (Region 1; Fig. 7). Meanwhile, the slow group displayed desynchronization, which descriptively appeared in the theta (Region 5; Fig. 7), alpha, and beta bands (Region 4; Fig. 7) during initial adaptation and the alpha and beta bands during initial postadaptation (Region 6; Fig. 7). Furthermore, fast adapters demonstrated enhanced desynchronization during early adaptation (Region 2; Fig. 7), which was notably absent in the slow group. The group difference in spectral power during early adaptation was significant, with fast adapter displaying decreased spectral power that descriptively appeared following left foot contact across delta (2–4 Hz), theta, and alpha bands (Region 7; Fig. 7).

In the bilateral anterior cingulate cortex, the fast group displayed increased spectral power during multiple conditions, whereas the slow group exhibited no significant differences from preadaptation. The fast group showed increased spectral power near the left anterior cingulate cortex during early adaptation (Region 1; Fig. 8) and initial postadaptation. Near the right anterior cingulate cortex, fast adapters demonstrated increased spectral power during early adaptation (Region 1; Fig. 9), initial postadaptation (Region 2; Fig. 9), and late postadaptation (Region 3; Fig. 9). Descriptively, these spectral fluctuations were most prominent in the delta and theta bands. There were no group differences near either of the anterior cingulate cortices.[Fig EN-NWR-0515-23F10]

The right visual cortex demonstrated a significant effect of condition on gait-related spectral power, but the left visual cortex showed none. Fast adapters exhibited both a negative (Region 1; Fig. 11) and positive (Region 2) change in spectral power during initial adaptation. This extended into early adaptation, with another two significant pixel clusters displaying a decrease (Region 3; Fig. 11) and increase (Region 4; Fig. 11) in spectral power. Descriptively, the desynchronizations were most evident in the alpha band, though some changes were observed within the theta and beta bands. The synchronizations for fast adapters were descriptively found within the beta and gamma (>30 Hz) bands. Conversely, slow adapters showed no significant spectral fluctuations during initial and early adaptation. Instead, we observed greater synchronization during late adaptation in the slow group (Region 6; Fig. 11). Compared with the fast group, the slow group exhibited less neural desynchronization during initial adaptation (Region 7; Fig. 11) and greater synchronization during late adaptation (Region 8; Fig. 11). Descriptively, fast adapters displayed greater alpha desynchronization during initial adaptation, and slow adapters exhibited greater alpha and beta synchronization approximately following left foot contact during late adaptation.

**Figure 12.**
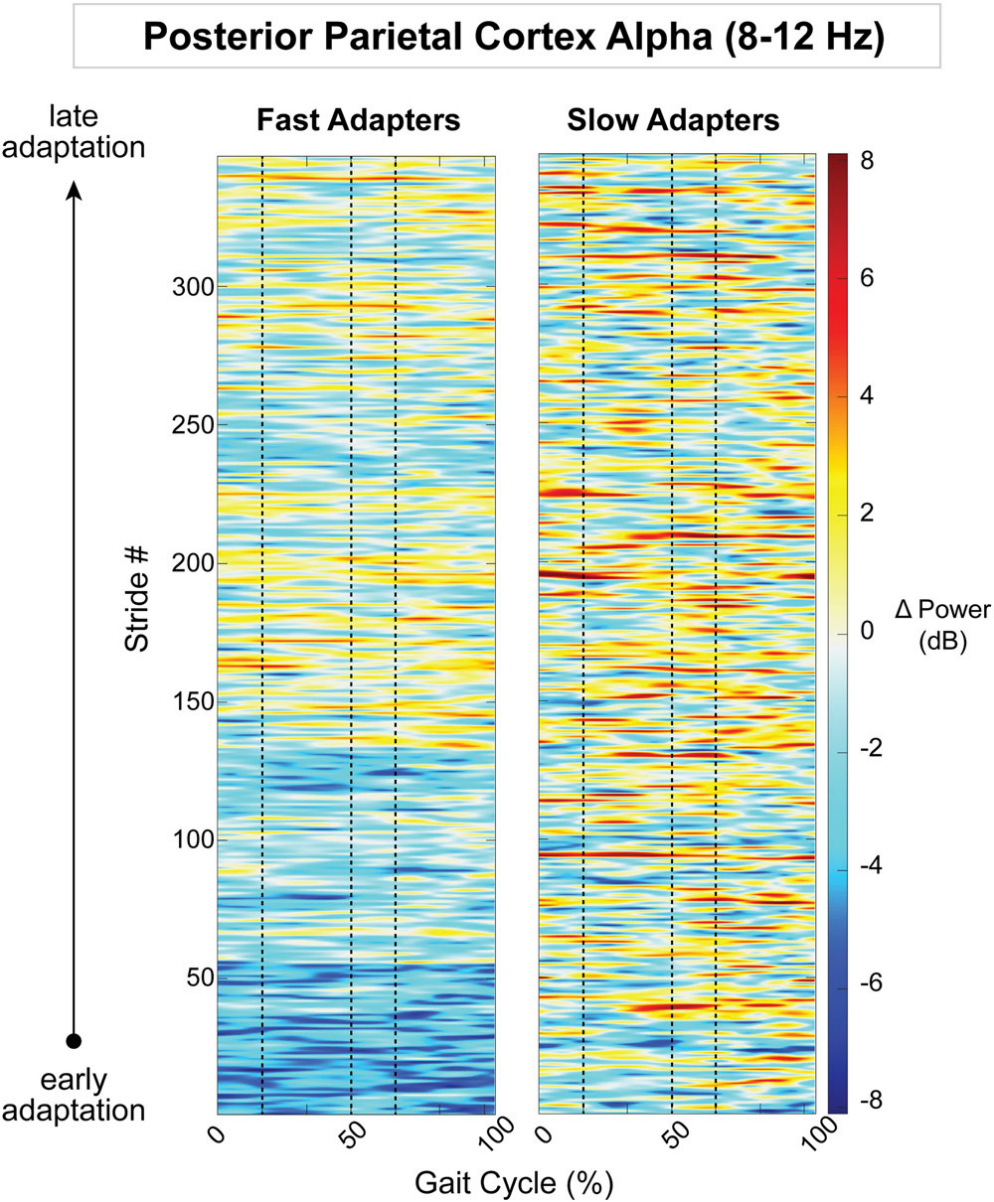
Group mean stride-by-stride variation in the posterior parietal cortex alpha (8–12  Hz) power across adaptation with respect to preadaptation. Data are truncated in length to match the participant who took the fewest strides during adaptation. The *x*-axis is the gait cycle, starting with the right foot contact at zero. Vertical dashed lines indicate the following gait events in order: left toe-off, left foot contact, and right toe-off.

**Figure 13.**
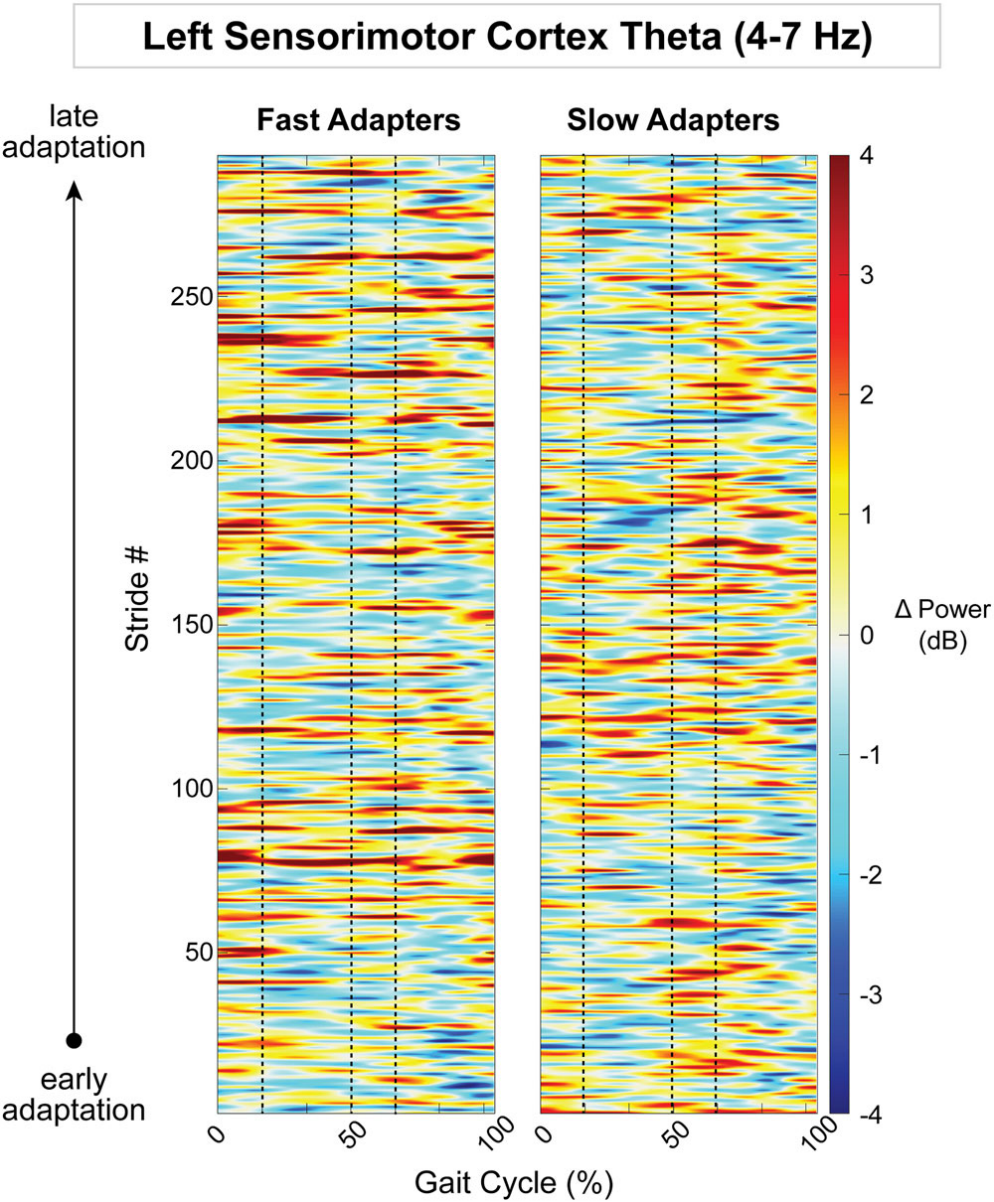
Group mean stride-by-stride variation in the left sensorimotor cortex theta (4–7  Hz) power across adaptation with respect to preadaptation. Data are truncated in length to match the participant who took the fewest strides during adaptation. The *x*-axis is the gait cycle, starting with the right foot contact at zero. Vertical dashed lines indicate the following gait events in order: left toe-off, left foot contact, and right toe-off.

**Figure 14.**
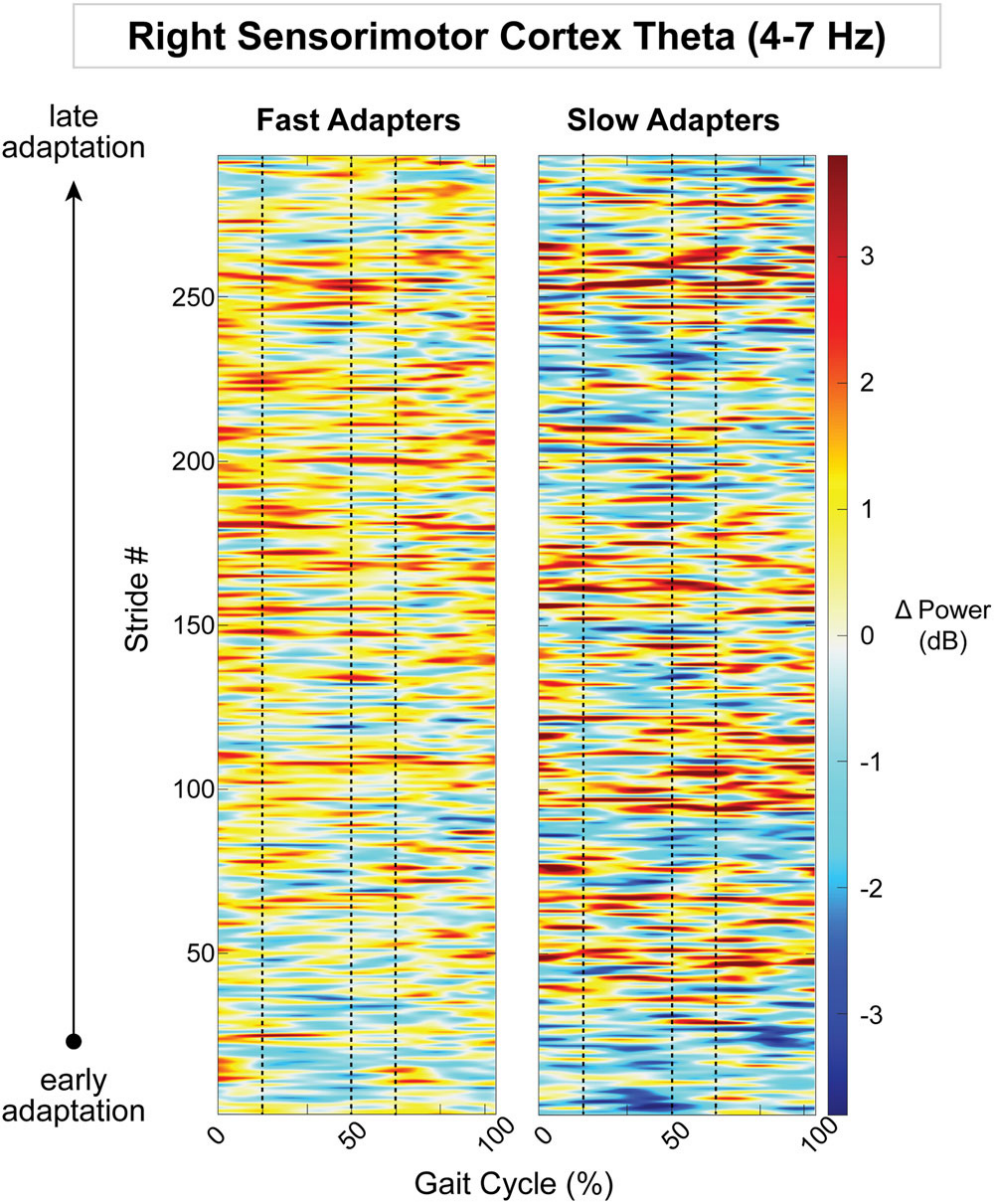
Group mean stride-by-stride variation in the right sensorimotor cortex theta (4–7 Hz) power across adaptation with respect to pre-adaptation. Data are truncated in length to match the participant who took the fewest strides during adaptation. The x-axis is the gait cycle, starting with the right foot contact at zero. Vertical dashed lines indicate the following gait events in order: left toe-off, left foot contact, and right toe-off.

**Figure 15.**
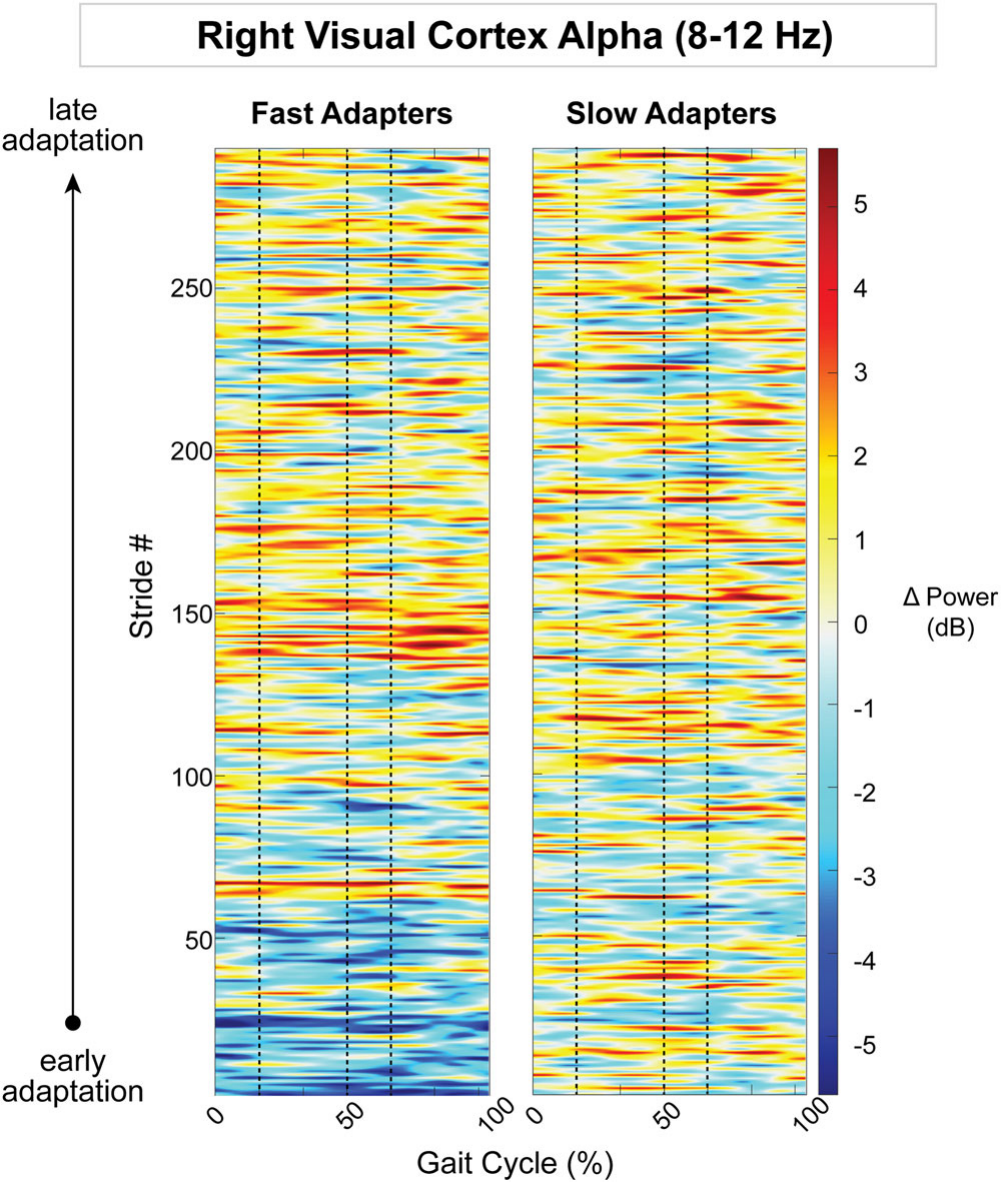
Group mean stride-by-stride variation in the right visual cortex alpha (8–12  Hz) power across adaptation with respect to preadaptation. Data are truncated in length to match the participant who took the fewest strides during adaptation. The *x*-axis is the gait cycle, starting with the right foot contact at zero. Vertical dashed lines indicate the following gait events in order: left toe-off, left foot contact, and right toe-off.

Because the posterior parietal, bilateral sensorimotor, and right visual cortices displayed an effect of group on gait-related spectral power, we descriptively examined how spectral power changed on a stride-by-stride basis over the course of adaptation. Posterior parietal alpha desynchronization was observed in group-averaged strides from fast adapters during early adaptation (Fig. 12). This activity diminished over the course of adaptation, as step lengths became more symmetric. Slow adapters, on the other hand, did not exhibit any trends in alpha fluctuations throughout adaptation in the posterior parietal cortex (Fig. 12). Within the theta band, there were no obvious spectral patterns within the left sensorimotor cortex (Fig. 13); however, in the right sensorimotor cortex, slow adapters appeared to have more theta fluctuations than fast adapters (Fig. 14). In the right visual cortex, fast adapters displayed reduced alpha power during early adaptation compared with slow adapters (Fig. 15).

## Discussion

In line with our hypothesis, we found specific electrocortical spectral patterns that differed between the slow and fast adaptation groups, suggesting that this EEG activity represents neural correlates of gait adaptability. We uncovered multiple cortical regions with group differences in gait-related spectral power, namely, the posterior parietal, left sensorimotor, right sensorimotor, and right visual cortices. Specifically, our results reveal that alpha oscillations within the posterior parietal and primary visual cortices and theta oscillations in the sensorimotor cortex differ between slow and fast adapters. Notably, lower alpha power in the posterior parietal cortex during early adaptation was linked to quicker attainment of steady-state step length symmetry. Our results suggest that alpha oscillations within the posterior parietal may play a mechanistic role in enhancing locomotor adaptation, potentially by regulating multisensory integration and visuospatial attention.

Alpha oscillations in the posterior parietal cortex may play a role in visuospatial attention, sensory integration, and motor planning during locomotor adaptation. Alpha oscillations in the parietal cortex have been shown to be related to intersensory reorienting (Misselhorn et al., 2019) and obstacle avoidance during treadmill walking and running (Nordin et al., 2019). During task engagement, alpha power is reduced in task-relevant regions (Bazanova and Vernon, 2014). Visuospatial attention can optimize the sensorimotor system, potentially by regulating the responsiveness of neural populations in sensory regions (Ling et al., 2009).

Our findings emphasize that the posterior parietal cortex may play a key role in gait adaptation. We identified a component cluster near the superior posterior parietal cortex, a brain region that represents the lower body [summarized in Sereno and Huang (2014)]. When examining this brain region during the initial and early phase of adaptation—which we defined as strides 1–10 and 11–40, respectively—we found that fast adapters exhibited lower alpha power across the gait cycle near the posterior parietal cortex. When looking at the entire adaptation period, alpha desynchronization gradually diminished as fast adapters restored step length asymmetry; however, slow adapters showed no systematic change in posterior parietal alpha during adaptation. This means that slow adapters did not have a delayed response of alpha reduction; thus, they did not display strong alpha desynchronization at any point of adaptation.

Accumulating evidence supports the role of the posterior parietal cortex in gait adaptation. Wagner et al. (2016) used high-density EEG to investigate brain activity in neurotypical individuals as they adjusted their gait to auditory stepping cues (Wagner et al., 2016). Their investigation focused on a brief adaptation period of fewer than 20 steps, which only allows us to compare our results from initial and early adaptation to theirs. Wagner et al. (2016) found enhanced alpha and beta desynchronization near the left and right posterior parietal cortex during gait adaptation to auditory stepping cues with respect to pretempo shift. This desynchronization persisted for at least 15 steps post-tempo shifts. The persistence and frequency range of spectral fluctuations we saw in the fast adaptation group near the posterior parietal cortex align with the results of Wagner et al. (2016). Later, Hinton et al. (2019) hypothesized that the posterior parietal cortex would be active during split-belt walking (Hinton et al., 2019). They found that peak glucose uptake near the posterior parietal cortex was greater during continuous split-belt treadmill adjustment compared with a tied-belt treadmill, indicating that the posterior parietal cortex is associated with gait adaptation (Hinton et al., 2019). Young et al. (2020) used transcranial magnetic stimulation to confirm that the posterior parietal cortex is indeed involved in split-belt adaptation (Young et al., 2020). Our preceding study, detailed in Jacobsen and Ferris (2023), examined the entire study population (i.e., both adaptation groups combined) and found that initial and early stages of split-belt adaptation were associated with alpha and beta desynchronization in the posterior parietal cortex. Here we demonstrated that the slow adaptation group lacked alpha fluctuations during adaptation, suggesting that the posterior parietal alpha desynchronization observed in Jacobsen and Ferris (2023) and Wagner et al. (2016) may be driven by individuals who adapt their gait more quickly.

Our investigation of alpha oscillations in the posterior parietal cortex during gait adaptation reveals nuanced differences between fast and slow adapters, shedding light on the cognitive mechanisms underlying sensorimotor integration and motor planning in response to locomotor challenges. Because fast adapters had more alpha suppression in the posterior parietal cortex during early adaptation than slow adapters, greater alpha suppression may reflect that more cognitive resources were being used to integrate sensory information and facilitate the search for adaptive leg movement solutions to counteract the difference in belt speeds. Participants with greater alpha suppression were possibly better at directing covert attention to the right sensory streams and integrating this information, allowing them to reduce kinematic errors at a faster rate.

It is unclear if posterior parietal alpha fluctuations are tonic or modulated by the gait cycle during gait adaptation. Wagner et al. (2016) found that alpha desynchronization peaked just prior to contralateral heel strikes, whereas we found that posterior parietal alpha desynchronization occurred throughout the gait cycle. Differences in the timing of desynchronization within the gait cycle may be attributed to our singular posterior parietal cluster located closer to the midline, potentially combining lateralized effects, whereas Wagner et al. (2016) identified bilateral clusters in the posterior parietal cortex. As gait is cyclic with constant motor activity, it is unclear whether alpha desynchronization is related to multiple overlapping sensorimotor events or, contrarily, a tonic cognitive state that is not modulated by the gait cycle. Moreover, task variations also likely contributed to some discrepancies in results, with our study involving mechanical perturbations and (Wagner et al., 2016) using auditory cues, the latter requiring more explicit adaptation. A future EEG study that uses a single-leg adaptation paradigm (e.g., introducing a force via a cable that pulls only one leg; Blanchette and Bouyer, 2009; Kitatani et al., 2022) might differentiate tonic changes versus gait-related changes in the posterior parietal cortex.

Slow adapters exhibited greater sensorimotor theta power compared with fast adapters at the onset of gait adaptation, suggesting potential differences in the perception of the physical gait perturbation. Evidence suggests that EEG theta power, or a comparable decrease in event-related potentials, is associated with physical loss of balance (Sipp et al., 2013; Payne and Ting, 2020) or postural threat (Goel et al., 2018; Peterson and Ferris, 2018; Payne and Ting, 2020; Solis-Escalante et al., 2021). Furthermore, the results of Wager et al. (2016) show that theta increases near the central midline during gait adaptation to auditory stepping cues. These studies highlight the role of theta oscillations in tasks that challenge gait and/or balance stability. Given that slow adapters exhibited higher theta power than fast adapters in the left and right sensorimotor cortex during initial and early adaptation, respectively, it is possible that slow adapters had more difficulty with predicting the gait perturbations. This increased theta power may reflect greater sensory mismatch between predicted and actual body kinematics. Overall, these findings suggest that reduced theta power in the sensorimotor cortex may indicate smaller sensory prediction errors, facilitating faster gait adaptation.

Group differences within the sensorimotor cortex varied across conditions depending on the hemisphere, indicating lateralization of electrocortical activity. Previous research on balance loss revealed greater theta synchronization in the left sensorimotor area compared with the right, irrespective of the direction of balance disruption (Sipp et al., 2013). Similarly, in a more recent study, we observed that theta synchronization occurs earlier in the left than the right sensorimotor area in response to walking perturbations (Peterson and Ferris, 2018). These findings suggest that the left sensorimotor cortex may be more sensitive to balance loss, which could potentially explain why we observed differences between split-belt adaptation groups only in the left sensorimotor cortex (Region 3; Fig. 6) during initial adaptation. Our results may be attributed to the dominance of the left hemisphere in right-handed individuals during various skilled movements (Serrien et al., 2006), further supporting that participants’ footedness may contribute to asymmetrical cortical outcomes (Packheiser et al., 2020).

We did not observe any differences in sensorimotor beta power between slow and fast adapters. Beta desynchronization in sensorimotor regions has been posited to reflect a change from the status quo motor pattern (Engel and Fries, 2010). Results from discrete upper-limb tasks have suggested that this beta suppression also indicates the motor system's readiness to explore new motor patterns and deviate from the status quo (Tan et al., 2016). Based on this evidence, one might expect fast adapters to have stronger beta desynchronization, as they are more willing to search for a new gait pattern. However, we did not observe any group differences in beta power within the sensorimotor cortex. A major difference between our study and these other studies is that we used a continuous whole-body task, whereas the other studies used discrete upper-limb tasks. Walking requires rhythmic activation from many muscles. There is no distinct movement on and off phase, which is what many upper-limb studies used to interpret movement-related beta activity. Future sensorimotor adaptation studies could use continuous tasks that require multiple limbs to explore neural predictors of adaptation.

Although visual alpha oscillations can have multiple functions (Clayton et al., 2018), its role in gait adaptation is unclear. Vision plays an important role in posture and gait stability. Worse visual spatial ability has been associated with increased double support phase variability in older adults (Martin et al., 2013), demonstrating the importance of visuospatial processing for gait stability. Although participants walked on a treadmill while looking straight ahead, vision remains crucial for the vestibular–ocular system to perceive the body's orientation in the surrounding environment (Lee, 1980; Perry et al., 2001) and sustain dynamic stability during walking (Hallemans et al., 2010). Fast adapters had lower alpha power in the right visual cortex compared with slower adapters during initial adaptation, which could be indicative of better visuospatial processing. Surprisingly, there were also significant differences in alpha and low beta activity between groups during late adaptation. Fast adapters had no gait-related spectral functions during late adaptation (with respect to preadaptation). Contrarily, slow adapters exhibited a significant increase in alpha and beta power, with respect to preadaptation, during double support of late adaptation. This activity occurred in the visual cortex contralateral to the foot contacting the slow belt. It is unclear why slow adapters have enhanced alpha and beta power in the right visual cortex during late adaptation compared with fast adapters. Alpha and beta activities do not appear to be related to gait stability, as step width variability was not significantly different between groups. Physiologically, visual alpha desynchronization is interpreted as increased cortical excitability (Romei et al., 2008a,b). In the context of visual spatial attention, alpha synchronization would indicate that cortical excitability is decreased during slow belt (0.6 m/s) double support compared with tied-belt preadaptation (0.9 m/s) for slow adapters. One possible explanation is that alpha oscillations serve to gate neural activity downstream of the visual cortex (i.e., modulate feed-forward flow to parieto-occipital areas; Zhigalov and Jensen, 2020). The alpha fluctuations seen at the beginning (fast adapters) and end of adaptation (slow adapters) also may represent fundamentally different neurophysiological processes given that the former was widespread across the gait cycle and the latter was more focal in time. It is possible that slow and fast adapters have different adaptation strategies that require varying levels of engagement from the visual cortex.

There were also some conditions and brain regions that displayed no group differences. We only found group effects during adaptation subconditions, with no significant differences between groups emerging during the post-adaptation period. This is not surprising given that there was no difference in deadaptation of step length asymmetry between groups. Additionally, there were no differences in anterior cingulate spectral power between slow and fast adapters, though fast adapters were the only group to display a significant change from preadaptation. Given the association between midfrontal electrocortical dynamics, error awareness (Ficarella et al., 2019), and balance ability (Sipp et al., 2013; Payne and Ting, 2020; Solis-Escalante et al., 2021; Stokkermans et al., 2022), it is surprising there were no significant group differences in this brain region.

Our study has several implications for clinical research and applications. By uncovering the distinctive brain dynamics associated with individual variation in gait adaptation, it lays the groundwork for tailored rehabilitation protocols. The identification of electrocortical activity related to gait adaptation performance suggests valuable neural predictors of treatment response in clinical settings may exist. For instance, a stroke patient's EEG signature might reveal their suitability for specific training regimens. Moreover, incorporating neurofeedback into standard rehabilitation protocols can enable real-time adjustments, potentially improving outcomes. Furthermore, insights into the role of alpha oscillations within the posterior parietal and visual cortices during gait adaptation offer promising avenues for novel therapeutic interventions. Techniques such as transcranial magnetic stimulation or neurofeedback training could be explored to modulate alpha oscillations, potentially enhancing motor learning and adaptation in clinical populations. Additionally, our study delineates not only the cortical regions implicated in adaptation but also their dynamics across the gait cycle. This knowledge could inform studies aiming to optimize motor adaptation through precisely timed brain stimulation interventions, as the timing of such interventions emerges as a critical determinant of efficacy (Weightman et al., 2022). Overall, these findings provide a basis for future clinical research, offering the potential to enhance the effectiveness, precision, and personalization of rehabilitation methods for individuals with gait impairments.

### Limitations

There were limitations to our research study. First, there may be other variables besides step length asymmetry (e.g., other measures of gait stability and muscle activity) that we did not quantify that explain the difference in electrocortical activity between groups. Next, the grouping method required an arbitrary selection of parameters to calculate the number of strides to reach steady-state step length asymmetry, namely, the number of SDs and number of strides to define the plateau. This method conflates the adaptation rate with the variability in the symmetry length asymmetry series over the last 30 strides. For instance, this method may indicate a longer time to reach steady-state for a step length asymmetry series with lower variability in the last 30 strides. Future studies that apply exponential models may be able to address this limitation (Rashid et al., 2020). Another limitation is that it is challenging to dissociate adaptation and voluntary corrections during walking. Both processes can occur in parallel. The accelerated adaptation may be explained by unchanged implicit adaptation combined with a distinct voluntary correction process (Roemmich et al., 2016). However, we tried to minimize sensory information that could be used for voluntary corrections, such as view of the belts. Additionally, electrophysiological activity from other brain structures, such as the cerebellum, may be driving group differences in adaptation, but we cannot record these regions with EEG. Another limitation is that the visual cortex cluster likely has some residual neck muscle activity contamination, so broadband activity in the gamma range (>30 Hz) may not reflect brain sources. For this reason, we did not discuss activity in this frequency range. Furthermore, using high-density EEG for cortical source localization offers a spatial resolution of ∼1–2 cm (Sohrabpour et al., 2015; Seeber et al., 2019). Consequently, one should exercise some caution when interpreting anatomical locations. Nevertheless, our findings provide a reasonable estimate of specific regions involved in gait adaptation. Lastly, given the lack of direct neural evidence related to individual differences during continuous sensorimotor adaptation tasks, our research should be taken as exploratory.

## Conclusion

Overall, our study marks a significant advancement in understanding the neural correlates of gait adaptation. Unlike previous research that focused on upper-limb sensorimotor adaptation, our study uniquely demonstrates that electrocortical activity differs between individuals who adapt their gait quickly versus more slowly. We identified distinct patterns of alpha oscillations within the posterior parietal cortex, theta oscillations in the bilateral sensorimotor cortex, and alpha/beta oscillations within the right primary visual cortex, which differed between slow and fast adapters. Notably, the group effect on posterior parietal spectral power suggests that reduced alpha power during early adaptation may play a pivotal role in facilitating faster locomotor adaptation. These findings align with the role of the posterior parietal cortex in sensorimotor adaptation and its importance in spatial attention, sensory integration, and movement planning. Additionally, differences in theta oscillations within the sensorimotor cortex may reflect greater sensory prediction errors within the slow adaptation group. Furthermore, our observations regarding visual cortex activity suggest potential implications for visuospatial processing during gait adaptation. Results from this exploratory analysis provide a basis for more hypothesis-driven research to elucidate distinct brain dynamics responsible for individual variation in gait adaptation. Despite certain limitations, this research advances our understanding of neural correlates of locomotor adaptability, which may ultimately inform strategies for enhancing motor rehabilitation and performance.

## Synthesis

Reviewing Editor: David Schoppik, New York University Decisions are customarily a result of the Reviewing Editor and the peer reviewers coming together and discussing their recommendations until a consensus is reached. When revisions are invited, a fact-based synthesis statement explaining their decision and outlining what is needed to prepare a revision will be listed below. The following reviewer(s) agreed to reveal their identity: Nadine Jacobsen.

Page numbers refer to the page within the merged pdf.

Major

The data bears similarity to the data analyzed in Jacobsen &Ferris (2023). If this is true, the final version of the manuscript will need to specify that data has been previously analyzed and in what contexts. The same holds for figure 1 and 2; please mention that these are adapted figures in the final version.

Some gait adaptation studies are mentioned in the introduction (Hinton et al., 2019; Jacobsen &Ferris, 2023; Wagner et al., 2016, 2019). Could the authors discuss their findings regarding these studies? Please also consider citing / discussing Khajuria A and Joshi D, 10.1016/j.bbe.2022.01.003 and Goel R, Ozdemir RA, Nakagome S, Contreras‑Vidal JL, Paloski WH, Parikh PJ, 10.1007/s00221-018-5284-5

The authors state that there is a lack of previous studies examining the differences in electrocortical activity between slow and fast learners during walking, which would provide a basis for forming a hypothesis. However, the hypothesis of the present study is still missing in the introduction.

The authors state that "electrocortical activity is correlated with adaptation rate"(p.21). We did not spot this analysis in the manuscript but would be very interested to see whether e.g. alpha power and adaptation rate indeed correlate. Maybe alpha power could be even used to predict adaptation performance?

Jacobsen &Ferris (2023, Figure 13) observed an alpha power decrease during initial and early adaptation at a cluster located close to the posterior parietal cortex, too. Could the authors comment on this in the discussion? Do they think that result could be driven by fast adapters? Moreover, the decrease in alpha power compared to baseline at the posterior parietal cortex seems to be not modulated by the gait cycle. Could they comment on whether they assume this is a sensible way to capture the phenomenon or whether they would choose a different approach in a potential future study?

Another potentially interesting analysis would be looking at the EEG activity during the post-adaptation period for fast and slow adapters (see e.g. Jacobsen &Ferris, 2023). Maybe patterns emerging during adaptation repeat.

Finally, the translation of the present findings into clinical applications is not clear. The article would benefit if the authors could explain this point in the Discussion section.

Minor

Introduction

p.5 "Variations in adaptation rate [...]" Please add a source. The authors could consider further highlighting why we would like to improve our understanding of cortical activities associated with fast/slow gait adaptation.

p.5 "One reason for this is that traditional brain imaging techniques like functional magnetic resonance imaging require participants to be stationary" As EEG is much older than MRI the authors may consider dropping "traditional".

p. 5 "This study aims to enhance our understanding of the neural correlates of gait adaptation [...]" We would move this sentence after the description of EEG methods and findings just before "Therefore we focused [...]". Please also include findings from Jacobsen &Ferris (2023) in this paragraph.

p. 5 "Therefore, we focused our time-frequency analysis on the theta, alpha, and beta bands." If this is the case, why do the authors show EEG activity in the gamma range (Figure 5-11)?

p.5 typo "We considered this as an exploratory study to determine if there were significant

*differences* in electrocortical activity between fast and slow gait adapters."

Methods

Overall, thank you very much for including many details on your (EEG) preprocessing and analysis pipeline!

Did the authors assess whether the belt speeds were comfortable for the participants?

p.7 EMG "Neck electromyography (EMG) was recorded from the left and right sternocleidomastoid, splenius capitis and trapezius to isolate muscle activity and enhance EEG data quality" The EMG recording itself does not improve EEG data quality. Maybe the authors could rephrase and refer the reader to the "EEG analysis" section for how EMG was used to identify artifacts?

p. 7 Dual layer setup: Please include BioSemi's geographic location. The authors could consider providing RRIDs to facilitate re-identification of equipment and software.

p.7 "This dual-electrode system exhibited a twofold enhancement in signal-to-noise ratio in a phantom head simulation experiment (redacted reference)." Please include another source for this claim.

p.8 "All scalp electrode offsets were at or below 20 mV before recording." If the authors also have impedance measurements, could these be included here?

p. 9 "[...] down sampled to *xx Hz* aligned with the EEG sampling rate." Including the target frequency may facilitate reading although it is technically repeated information.

p.9 "Step length asymmetry was normalized across subjects by subtracting participants' average step-length asymmetry of pre-adaptation walking at 0.9 m/s." Was the normalization done within subject or with average pre-adaptation step-length asymmetry across participants?

p. 9 For Different parts of the adaptation process a different number of strides (e.g. initial and early adaptation 10 and late adaptation 30 strides). Could the authors explain what the reasoning behind his was? Moreover, I assume the division is the same as in Jacobsen and Ferris (2023). Could this reference be added, please.

p.9 typo "The head model*s* were..."

p.11 "The EEG processing pipeline was executed using Matlab 2022a (MathWorks, Natick, MA), EEGLAB (v2022.0), open-source software such as EEGLAB (Delorme &Makeig, 2004)" EEGLAB is mentioned twice. If any other open source toolbox was used I would kindly ask the authors to include a reference and version as well.

p.11 "[..] individually re-referenced using the average across channels after the temporary removal of bad channels (> 3 SD)." How many channels were on average rejected based on this criterion?

p. 11 "Bad channels were excluded based on statistical criteria: probability, standard deviation, and kurtosis". Could the authors add the thresholds for all criteria?

p.11 clean:artifacts: Please include the version and the used parameters.

p.12 Please include some information on the way they performed the frequency decomposition (method and specs)

p.13 "We quantified gait-related spectral..." This and the next paragraph describe the statistical evaluation of the EEG and behavioral data. I would insert a respective heading before this sentence. Could you also incorporate which test statistic was used for the cluster-based permutation test? How many tests were calculated and was the alpha-error/p-vale corrected accordingly?

p.13 "To maintain consistency with EEG statistical testing [...]" We assume consistency was not the only reason to opt for cluster-based permutation testing. Could the authors also highlight their other reasons?

p.14 "*Descriptively* there were no large differences in age, body mass index, or sex distribution between groups." Or did we overlook a statistical evaluation?

Results

Whenever possible, please include effect sizes see e.g. https://www.fieldtriptoolbox.org/example/effectsize/#statistical-inference-using-a-cluster-based-permutation-test

p. 16 "During initial and early adaptation, fast adapters had lower alpha power near the posterior parietal cortex than slow adapters [...]" To an unaware reader this may sound like the cluster-based permutation test allows us to locate (time/frequency) the difference between the groups (see https://www.fieldtriptoolbox.org/faq/how_not_to_interpret_results_from_a_cluster-based_permutation_test/). The authors could consider rephrasing this sentence to underline that the location of the difference is descriptive.

p.17 "We found specific electrocortical spectral patterns that *differed between* the slow and fast adaptation groups, suggesting that this EEG activity represents neural predictors of gait adaptability." Differentiated and predictor may suggest that a classification based on EEG activity was performed while participants were divided into two groups based on their gait performance. I would suggest a slight rephrase to reflect this, possibly also on other points in the discussion.

p.17 "We uncovered two key cortical regions, the posterior parietal, and *the right visual cortex*" No significant difference in the left visual cortex was found, was it?

p.17 paragraph "The posterior parietal cortex..." until "The component cluster we identified..." Since the posterior parietal cortex was an area of interest a priori I suggest moving this section into the introduction and combining the remainder of the paragraph with the following one.

p.19 typo "One possible explanation *is* that"

The authors highlight the potential role of visual information for gait adaptation. How does this relate to the current study in which participants were instructed to not look down (as mentioned in the limitations) and the scenery did not change as they were walking on a treadmill? Was there much altered visual input accompanying the gait adaptation?

We request that the authors add a statement to the paper confirming whether, for all experiments, they have reported all measures, conditions, data exclusions, and how they determined their sample sizes. The authors should, of course, add any additional text to ensure the statement is accurate. This is the standard reviewer disclosure request endorsed by the Center for Open Science [see http://osf.io/hadz3].

## Author Response

eNeuro Rebuttal Major The data bears similarity to the data analyzed in Jacobsen &Ferris (2023). If this is true, the final version of the manuscript will need to specify that data has been previously analyzed and in what contexts. The same holds for figure 1 and 2; please mention that these are adapted figures in the final version.

We have added a description of the data that have been previously published in Jacobsen &Ferris (2023) in our Introduction section (lines 131-140). We have also added a note to the figure captions (1&2) that they are adapted from Jacobsen &Ferris (2023).

Some gait adaptation studies are mentioned in the introduction (Hinton et al., 2019; Jacobsen &Ferris, 2023; Wagner et al., 2016, 2019). Could the authors discuss their findings regarding these studies? Please also consider citing / discussing Khajuria A and Joshi D, 10.1016/j.bbe.2022.01.003 and Goel R, Ozdemir RA, Nakagome S, ContrerasVidal JL, Paloski WH, Parikh PJ, 10.1007/s00221-018-5284-5 We have discussed our findings in the context of Jacobsen &Ferris (lines 528-534), Hinton et al. (2019; lines 522-526), Wagner et al. (2016; lines 514-522, 532-534, 545-556, 804-805), and Goel et al. (2018; lines 562-564).

We did not discuss the other two studies the reviewer mentioned. We did not find a cluster near the medial pre-frontal cortex as Wanger et al. (2019) did, so we cannot compare our result to this study. Khajuria et al. investigated sensorimotor alpha power in individuals with and without leg amputation during voluntary postural sway. They found that postural instability led to alpha and beta desynchronization near the bilateral sensorimotor cortex in participants with intact limbs (Khajuria et al., 2022). Consistent with control group in Khajuria et al., both fast and slow adapters exhibited heightened alpha desynchronization in the right sensorimotor cortex during initial adaptation, a period marked by heightened gait instability. However, in the left sensorimotor cortex, slow adapters didn't have any significant spectral fluctuations during adaptation. The group differences appear to primarily occur within the theta band. Perhaps the differences in gait stability between the slow and fast groups was not great enough to evoke large changes in alpha power, as it did with amputees vs. controls. However, we can't make this comparison as Khajuria et al. examined a clinical population and we do not have measurements of gait stability to comment on if/how that affects the lack of differences in alpha and beta power between groups. Additionally, the event-related spectral perturbation plots in Khajuria et al. (2022) are not masked for significance, so it is unclear if the spectral fluctuations they show are significantly different from the baseline period. Therefore, it is difficult to comment on these results and we do not feel that it fits well within our discussion. However, we have added an additional paragraph that addresses group differences within the sensorimotor cortex within the context of other studies (including Goel et al. 2018, as suggested) focused on balance and gait perturbations (lines 560-573).

The authors state that there is a lack of previous studies examining the differences in electrocortical activity between slow and fast learners during walking, which would provide a basis for forming a hypothesis. However, the hypothesis of the present study is still missing in the introduction.

Given the lack of previous studies, we did not form specific hypotheses about spectral changes in specific brain regions, frequency bands, and points in the gait cycle. Overall, we hypothesized that spectral analyses would reveal differences in electrocortical activity in motor and sensory brain regions between fast adapters and slow adapters and have added this to the introduction (lines 140-142).

The authors state that "electrocortical activity is correlated with adaptation rate"(p.21). We did not spot this analysis in the manuscript but would be very interested to see whether e.g. alpha power and adaptation rate indeed correlate. Maybe alpha power could be even used to predict adaptation performance? We thank the reviewer for their suggestion and agree that this analysis would be insightful. Following the reviewer's recommendation, we have conducted an additional statistical analysis to determine if alpha power in the posterior parietal and right visual cortex is correlated with the number of strides to reach steady-state step length asymmetry (SLA). We averaged the alpha power (8-12 Hz) across the gait cycle for each adaptation condition and used a linear regression model to evaluate its correlation with the number of strides to reach steady-state SLA. We also did the same correlation analysis for theta power (4-7 Hz) in the sensorimotor cortex, give our most recent results in group differences approximately in the theta band. We found no correlation in all four of these models (all p>0.05).

Additionally, we explored the correlation between mean step length asymmetry and frequency band power (alpha in posterior parietal and right visual; theta in right and left sensorimotor). We only found one significant positive correlation (p = 0.012, adjusted R2 = 0.211), and that was between theta power and mean step length symmetry in the left sensorimotor cortex during early adaptation. The remaining linear regression models either did not demonstrate a linear relationship or had skewed residuals, indicating that linear models were not an appropriate fit given the data distribution.

A couple of potential explanations may account for the absence of a significant correlation between spectral power and the selected behavioral metrics. Primarily, the lack of observed effects could be attributed to the size of the binning window used. Our decision to average frequency band power across the entire gait cycle stemmed from a lack of a priori hypotheses regarding the temporal occurrence of spectral differences. However, this broad averaging window might obscure effects, especially for spectral fluctuations that are more focal in time. Narrowing the window based on preliminary findings could introduce bias into the correlation analysis. While alternative methods for selecting time and frequency windows exist, we posit that such endeavors would be more beneficial in studies boasting larger sample sizes per group and more refined hypotheses. Additionally, the absence of correlation could stem from the inadequacy of the chosen behavioral metric. It is plausible that metrics related to gait and postural stability might demonstrate stronger associations with spectral power than measures such as step length asymmetry.

Given the above reasons, we have not included the results of this correlation analysis in this manuscript. If you would like us to include it in the manuscript or in supplementary material, please let us know. Regardless, we have amended our conclusion to clarify that electrocortical activity differs between individuals who adapt their gait quickly versus more slowly, instead of saying it was correlated with adaptation rate (lines 688-690).

Jacobsen &Ferris (2023, Figure 13) observed an alpha power decrease during initial and early adaptation at a cluster located close to the posterior parietal cortex, too. Could the authors comment on this in the discussion? Do they think that result could be driven by fast adapters? Moreover, the decrease in alpha power compared to baseline at the posterior parietal cortex seems to be not modulated by the gait cycle. Could they comment on whether they assume this is a sensible way to capture the phenomenon or whether they would choose a different approach in a potential future study? We agree that the results from Jacobsen &Ferris could be driven by fast adapters and have added a comment on this in the discussion (531-534). We have also commented on the fact that alpha desynchronization does not appear to be modulated by the gait cycle, though our midline parietal cluster could be blurring lateralized effects (line 548-551). We have discussed our results in the context of Wanger et al. (2016) and added a recommendation on how a future study may attempt to differentiate tonic vs. gait-related changes in the posterior parietal cortex (lines 556-559).

Another potentially interesting analysis would be looking at the EEG activity during the post-adaptation period for fast and slow adapters (see e.g. Jacobsen &Ferris, 2023). Maybe patterns emerging during adaptation repeat.

We have added these conditions and created new figures. Similar patterns do repeat during the post-adaptation period.

Finally, the translation of the present findings into clinical applications is not clear. The article would benefit if the authors could explain this point in the Discussion section.

Thank you. We have added a paragraph to the discussion to explain the implications of our findings for clinical research and applications (lines 637-654).

Minor Introduction p.5 "Variations in adaptation rate [...]" Please add a source. The authors could consider further highlighting why we would like to improve our understanding of cortical activities associated with fast/slow gait adaptation.

Thanks for your suggestion. We have added additional citations demonstrating how adaptation rate is variable throughout different age groups in a neurotypical population (line 82). To better highlight the significance of this work, we have added to our introduction that gaining insight into the variability of brain activity among neurotypical individuals could enhance our ability to discern and characterize abnormal patterns of brain activity within clinical populations (line 87-101). p.5 "One reason for this is that traditional brain imaging techniques like functional magnetic resonance imaging require participants to be stationary" As EEG is much older than MRI the authors may consider dropping "traditional".

Thanks, we agree and have removed "traditional" (line 85). p. 5 "This study aims to enhance our understanding of the neural correlates of gait adaptation [...]" We would move this sentence after the description of EEG methods and findings just before "Therefore we focused [...]". Please also include findings from Jacobsen &Ferris (2023) in this paragraph.

We have moved this sentence to the methods and included findings from Jacobsen &Ferris (2023) (lines 131-137) p. 5 "Therefore, we focused our time-frequency analysis on the theta, alpha, and beta bands." If this is the case, why do the authors show EEG activity in the gamma range (Figure 5-11) We extend the range of the event-related spectral perturbation (ERSP) plots to give the reader a fuller picture of the extent of the spectral fluctuations. It also allows them to check for data quality-ERSPs with high power in low (delta/theta) frequencies or broadband spectral fluctuations in high (gamma) frequency ranges would indicate heavy motion and muscle artifact contamination, respectively. To avoid confusion, we have removed the gamma label from the plot so the reader is not drawn to it. However, we wish to keep the full 2-50 Hz range in the plots. p.5 typo "We considered this as an exploratory study to determine if there were significant *differences* in electrocortical activity between fast and slow gait adapters." We have modified this sentence, and this comment is no longer applicable.

Methods Overall, thank you very much for including many details on your (EEG) preprocessing and analysis pipeline! Thank you for your positive assessment of our data analysis! Did the authors assess whether the belt speeds were comfortable for the participants? We conducted pilot testing with participants of a similar age range to our study, testing various speeds to empirically determine the most comfortable one. p.7 EMG "Neck electromyography (EMG) was recorded from the left and right sternocleidomastoid, splenius capitis and trapezius to isolate muscle activity and enhance EEG data quality" The EMG recording itself does not improve EEG data quality. Maybe the authors could rephrase and refer the reader to the "EEG analysis" section for how EMG was used to identify artifacts? Thank you. We have revised this to say that the EMG is used to attenuate muscle artifacts within the EEG signal and refer the reader to the EEG analysis section (line 163-164). p. 7 Dual layer setup: Please include BioSemi's geographic location. The authors could consider providing RRIDs to facilitate re-identification of equipment and software.

The geographic location is included on line 166-167. We also added it to line 172 for redundancy. We have also added RRID numbers (e.g. line 172) p.7 "This dual-electrode system exhibited a twofold enhancement in signal-to-noise ratio in a phantom head simulation experiment (redacted reference)." Please include another source for this claim.

We have added the source for this claim (line 226). p.8 "All scalp electrode offsets were at or below 20 mV before recording." If the authors also have impedance measurements, could these be included here? We do not have that data available; BioSemi Active II provides electrode offsets, not direct impedance measurements. p. 9 "[...] down sampled to *xx Hz* aligned with the EEG sampling rate." Including the target frequency may facilitate reading although it is technically repeated information.

We have repeated the sampling rate to facilitate reading (line 172. p.9 "Step length asymmetry was normalized across subjects by subtracting participants' average step-length asymmetry of pre-adaptation walking at 0.9 m/s." Was the normalization done within subject or with average pre-adaptation step-length asymmetry across participants? Thank you. Normalization was done within subjects. We have clarified this in lines 217-219. p. 9 For Different parts of the adaptation process a different number of strides (e.g. initial and early adaptation 10 and late adaptation 30 strides). Could the authors explain what the reasoning behind his was? Moreover, I assume the division is the same as in Jacobsen and Ferris (2023). Could this reference be added, please.

We added a reference that used similar stride binning for the early and late adaptation (Roemmich &Bastian 2015). They used 5 strides for the initial adaptation, 25 strides for early adaptation, and 30 strides for late adaptation. We used 10 strides for the initial adaptation because of the high variability of the EEG data and instability following an abrupt perturbation. We wanted the remaining subconditions to have a consistent number of strides, so we chose 30 for both early and late adaptation. There is no gold standard for the number of strides used for each subcondition in split-belt walking, and anywhere from ~5-200 strides has been used previously in published studies. We have elaborated on our explaining of stride binning in the behavioral data analysis section (lines 245-248, 249-251). p.9 typo "The head model*s* were..." Thanks. We have corrected this (line 257) p.11 "The EEG processing pipeline was executed using Matlab 2022a (MathWorks, Natick, MA), EEGLAB (v2022.0), open-source software such as EEGLAB (Delorme &Makeig, 2004)" EEGLAB is mentioned twice. If any other open source toolbox was used I would kindly ask the authors to include a reference and version as well.

We have removed the duplicate. All other toolboxes/plug-ins used are cited in the text throughout the EEG analysis section (e.g. line 281, line 293). p.11 "[..] individually re-referenced using the average across channels after the temporary removal of bad channels (> 3 SD)." How many channels were on average rejected based on this criterion? We have amended that 4 {plus minus} 3.8 (mean {plus minus} SD) channels were removed based on this criterion (line 270) p. 11 "Bad channels were excluded based on statistical criteria: probability, standard deviation, and kurtosis". Could the authors add the thresholds for all criteria? We have added the thresholds for these criteria (lines 279-280) p.11 clean:artifacts: Please include the version and the used parameters.

We have added the version and mentioned that default parameters from cleanrawdata v2.91 were used (line 281) p.12 Please include some information on the way they performed the frequency decomposition (method and specs) We have added more details on time-frequency analysis (lines 312-313,315-318, 321). p.13 "We quantified gait-related spectral..." This and the next paragraph describe the statistical evaluation of the EEG and behavioral data. I would insert a respective heading before this sentence. Could you also incorporate which test statistic was used for the cluster-based permutation test? How many tests were calculated and was the alpha-error/p-vale corrected accordingly? We have added new topic sentences to these paragraphs for clarity (lines 326-327 and 360).

We have also added that the maximum sum of the cluster-level test statistic was used to solve the multiple comparisons problem and provided the function parameters (lines 339-340). We are unclear about what tests the reviewer is asking about ("how many tests were calculated"). For each cluster-based permutation test, an individual sample statistics (e.g., t-values) is calculated for each time-frequency point (our time-frequency matrix size was 95 x 105). These individual sample statistics are combined to form the cluster-level statistic. Within each cluster-based permutation test, the number of cluster-level statistical tests run is equal to the value specified for the number of permutations (cfg.numrandomization) to obtain the null distribution of the cluster-level statistic. We chose 10,000 permutations because a large number of randomizations improves the accuracy of the Monte Carlo approximation. The p-values are obtained via statcondfieldtrip() within EEGLAB, which calls Fieldtrip statistical functions (ft_statistics_montecarlo) that correct for multiple comparisons using the cluster-based test statistic (line 340-343). p.13 "To maintain consistency with EEG statistical testing [...]" We assume consistency was not the only reason to opt for cluster-based permutation testing. Could the authors also highlight their other reasons? We reorganized this paragraph to consolidate the reasons for selecting this method (lines 362-367). p.14 "*Descriptively* there were no large differences in age, body mass index, or sex distribution between groups." Or did we overlook a statistical evaluation? Thank you. We have clarified that this was a descriptive observation (line 380).

Results Whenever possible, please include effect sizes see e.g. https://www.fieldtriptoolbox.org/example/effectsize/#statistical-inference-using-a-cluster-based-permutation-test.

We have added a table to the supplementary material that provides all effect sizes (as measured by the maximum Cohen's d value), p-values, and 95% confidence intervals. p. 16 "During initial and early adaptation, fast adapters had lower alpha power near the posterior parietal cortex than slow adapters [...]" To an unaware reader this may sound like the cluster-based permutation test allows us to locate (time/frequency) the difference between the groups (see https://www.fieldtriptoolbox.org/faq/how_not_to_interpret_results_from_a_cluster-based_permutation_test/). The authors could consider rephrasing this sentence to underline that the location of the difference is descriptive.

Thank you. We agree that this is an important distinction and have tried to keep this in mind when writing the manuscript. We have revised our language in this paragraph to better distinguish statistical vs descriptive observations (lines 419-429). We have also added a note that cluster-based permutations tests do not establish significance of effect latency (e.g. a specific point in the gait cycle) or frequency (lines 345-348). p.17 "We found specific electrocortical spectral patterns that *differed between* the slow and fast adaptation groups, suggesting that this EEG activity represents neural predictors of gait adaptability." Differentiated and predictor may suggest that a classification based on EEG activity was performed while participants were divided into two groups based on their gait performance. I would suggest a slight rephrase to reflect this, possibly also on other points in the discussion.

Thank you. We have modified the discussion as suggested so it does not suggest groups were classified based on EEG activity (line 485-486 and line 684) p.17 "We uncovered two key cortical regions, the posterior parietal, and *the right visual cortex*" No significant difference in the left visual cortex was found, was it? Correct, we only observed differences in the right visual cortex. We were broadly stating the lobes in which we found spectral changes, but have amended this for clarity (line 487-489). p.17 paragraph "The posterior parietal cortex..." until "The component cluster we identified..." Since the posterior parietal cortex was an area of interest a priori I suggest moving this section into the introduction and combining the remainder of the paragraph with the following one.

Thanks for the suggestion. We have reorganized accordingly (p. 6 and remainder of paragraph moved line 504-505). p.19 typo "One possible explanation *is* that" Thank you. We have fixed this typo (line 620).

The authors highlight the potential role of visual information for gait adaptation. How does this relate to the current study in which participants were instructed to not look down (as mentioned in the limitations) and the scenery did not change as they were walking on a treadmill? Was there much altered visual input accompanying the gait adaptation? Although participants walked on a treadmill while looking straight ahead, vision remains crucial for the vestibular-ocular system to derive the orientation of the swaying body within the environment (Lee 1980, Perry et al. 2001) and sustain dynamic stability during walking (Hallemans et al. 2010). We have added this comment on line 603-605.

Visual input did not change in the same way as overground walking, but it was not entirely static either. If the participants had been blindfolded and truly had no visual input, we predict that electrocortical activity in the visual cortex would not differ between groups and participants would have much greater gait instability during split-belt adaptation.

We request that the authors add a statement to the paper confirming whether, for all experiments, they have reported all measures, conditions, data exclusions, and how they determined their sample sizes. The authors should, of course, add any additional text to ensure the statement is accurate. This is the standard reviewer disclosure request endorsed by the Center for Open Science [see http://osf.io/hadz3].

Thank you for your commitment to research transparency. We have added this statement and added text to ensure the statement is accurate (lines 145-146)
